# Pressed Cement-Free and Low-Cement Materials Based on Recycled Concrete Powder

**DOI:** 10.3390/ma19163441

**Published:** 2026-08-13

**Authors:** Oleh Bordiuzhenko, Leonid Dvorkin, Vadim Zhitkovsky

**Affiliations:** Department of Building Products Technology and Materials Science, National University of Water and Environmental Engineering, 33028 Rivne, Ukraine; l.i.dvorkin@nuwm.edu.ua (L.D.); v.v.zhitkovsky@nuwm.edu.ua (V.Z.)

**Keywords:** recycled concrete powder, concrete waste, pressed materials, cement-free composites, low-cement composites, thermal activation, structure formation

## Abstract

The fine powder fraction generated during concrete recycling is often regarded as a low-value by-product or used as a filler in cement-based materials. This study investigates recycled concrete powder (RCP) as the main component of pressed cement-free and low-cement mineral composites. The <0.14 mm fraction was obtained by crushing and sieving concrete waste. Cylindrical specimens were produced by semi-dry pressing at 20 MPa with a forming moisture content of 12–13% and cured under humid-air conditions. Four systems were studied: untreated RCP, thermally activated RCP, RCP with 2.5 wt.% Portland cement, and RCP with 5 wt.% Portland cement. Thermal activation was performed at 600 °C for 2 h. Compressive strength, bulk density, and water resistance coefficient were determined at 3, 7, and 28 days. At 28 days, compressive strength increased from 6.9 MPa for untreated RCP to 11.4 MPa for thermally activated RCP and 12.8 MPa for RCP with 5 wt.% cement, while the water resistance coefficient increased from 0.61 to 0.86. DTA/TGA analysis revealed thermal effects and mass-loss patterns consistent with the presence and evolution of hydrated and carbonate-containing phases. The results demonstrate that RCP can serve as a structure-forming component in pressed cement-free and low-cement materials.

## 1. Introduction

The reduction in Portland cement consumption and the high-value reuse of mineral waste are among the key challenges in the development of low-carbon construction materials. Cement production remains highly energy- and resource-intensive and is associated with a significant share of global anthropogenic CO_2_ emissions [1]. Therefore, modern strategies for sustainable binders and mineral composites increasingly focus on lowering the clinker factor, using alternative supplementary cementitious materials and developing regenerated binding systems from industrial- or construction-related solid wastes [2,3,4]. In parallel, the accumulation of construction and demolition waste has become a major environmental and technological issue. Concrete waste forms a substantial part of this waste stream and its rational recycling can reduce landfill disposal, preserve natural aggregates and create secondary mineral resources for new construction materials [5,6,7].

The most common route for recycling concrete waste is its crushing and classification into recycled aggregates for concrete, road bases, backfills and other construction applications. However, the production of recycled aggregate is inevitably accompanied by the formation of fine and ultrafine fractions. These fractions are often difficult to reuse as conventional aggregates because of their high content of old cement paste, variable composition, increased water demand and high specific surface area. In the literature, related materials are also referred to as waste concrete powder (WCP), recycled concrete fines (RCF), or waste hardened cement paste powder. However, the term recycled concrete powder (RCP) is used consistently throughout this manuscript to denote powder obtained by crushing and classifying hardened concrete waste. Where particle size is relevant, the corresponding fraction is specified explicitly; the <0.14 mm fraction is referred to as ultrafine RCP. Recent reviews and experimental studies have shown that RCP can no longer be considered only as an inert waste product; depending on its origin, fineness and processing method, it may exhibit filler, nucleation and partial cementitious effects [8,9,10,11].

Previous studies have mainly investigated RCP as a partial cement replacement, supplementary cementitious material, sand replacement, or modifier of Portland-cement-based systems [12,13,14,15,16,17,18,19]. Its effects in these systems are associated with particle packing, nucleation, and the residual reactivity of cementitious phases, which may be enhanced by carbonation, thermal activation, chemical–thermal treatment, or combined activation with other mineral additives [8,9,10,11,14,20,21,22,23,24]. Because RCP originates from a previously hardened composite, its composition and performance depend strongly on the age, composition, and service history of the parent concrete, as well as on carbonation, adhered mortar content, particle-size distribution, crushing intensity, and subsequent treatment [25].

Despite the significant progress in the use of RCP in cement-based materials, most studies still consider it as an additive to a Portland cement system. In this approach, RCP partially replaces cement or sand, but the main binding function remains associated with newly introduced Portland cement. A less investigated route is the use of recycled concrete powder as the main component of a pressed mineral composite. Such an approach is fundamentally different from conventional cement replacement because the powder is not only an auxiliary component but also the main mineral matrix. Material development is then governed by compaction, interparticle contact development, residual hydration and secondary crystallization processes occurring in a limited pore space.

Pressure-assisted recycling of hardened cementitious materials offers a promising route for producing compacted products from concrete waste and industrial by-products [26,27,28]. Pressing increases packing density, reduces intergranular porosity, and promotes interparticle contact; its effectiveness depends on particle-size distribution, morphology, friction, moisture content, and applied pressure [29,30,31]. Subsequent hardening may involve capillary and surface interactions, residual hydration, dissolution–precipitation, and crystallization in interparticle zones [32]. Although related mechanisms have been considered for other low-clinker and chemically activated binders [33,34,35], their combined role in pressed cement-free and low-cement materials based primarily on RCP remains insufficiently understood.

Despite the progress achieved in pressure-assisted recycling of cementitious waste, the ability of RCP itself to act as the principal reactive matrix of pressed cement-free and low-cement materials remains insufficiently established. In particular, the combined effects of RCP origin, forming moisture, pressing pressure, curing conditions, particle-size composition, thermal activation, and small Portland cement additions have not been systematically considered within a single experimental framework. The relationship between these processing factors, the resulting mechanical and water-resistance properties, and the thermal evidence of phase transformations also requires further clarification.

Accordingly, this study tests the hypothesis that ultrafine RCP, when compacted at an appropriate moisture content and pressure and subsequently cured under humid-air conditions, can develop a load-bearing and water-resistant structure with no Portland cement or with only a small cement addition. This behaviour is expected to result from the combined effects of mechanical densification, development of interparticle contacts, residual hydration, and dissolution–precipitation processes involving cement paste residues; thermal activation or limited Portland cement addition may further enhance these processes. To test this hypothesis, the effects of concrete waste origin, forming moisture, pressing pressure, curing regime, thermal activation, particle-size composition, and Portland cement additions of 2.5 and 5 wt.% were evaluated using compressive strength, bulk density, water resistance, experimental–statistical modelling, and DTA/TGA analysis. The original contribution of the study lies in the integrated evaluation of RCP as the principal mineral matrix of pressed cement-free and low-cement composites, rather than as a filler, aggregate replacement, or supplementary cementitious component in a conventional Portland cement system.

## 2. Materials and Methods

### 2.1. Raw Materials and Preparation of Recycled Concrete Powder

**Figure 1:**
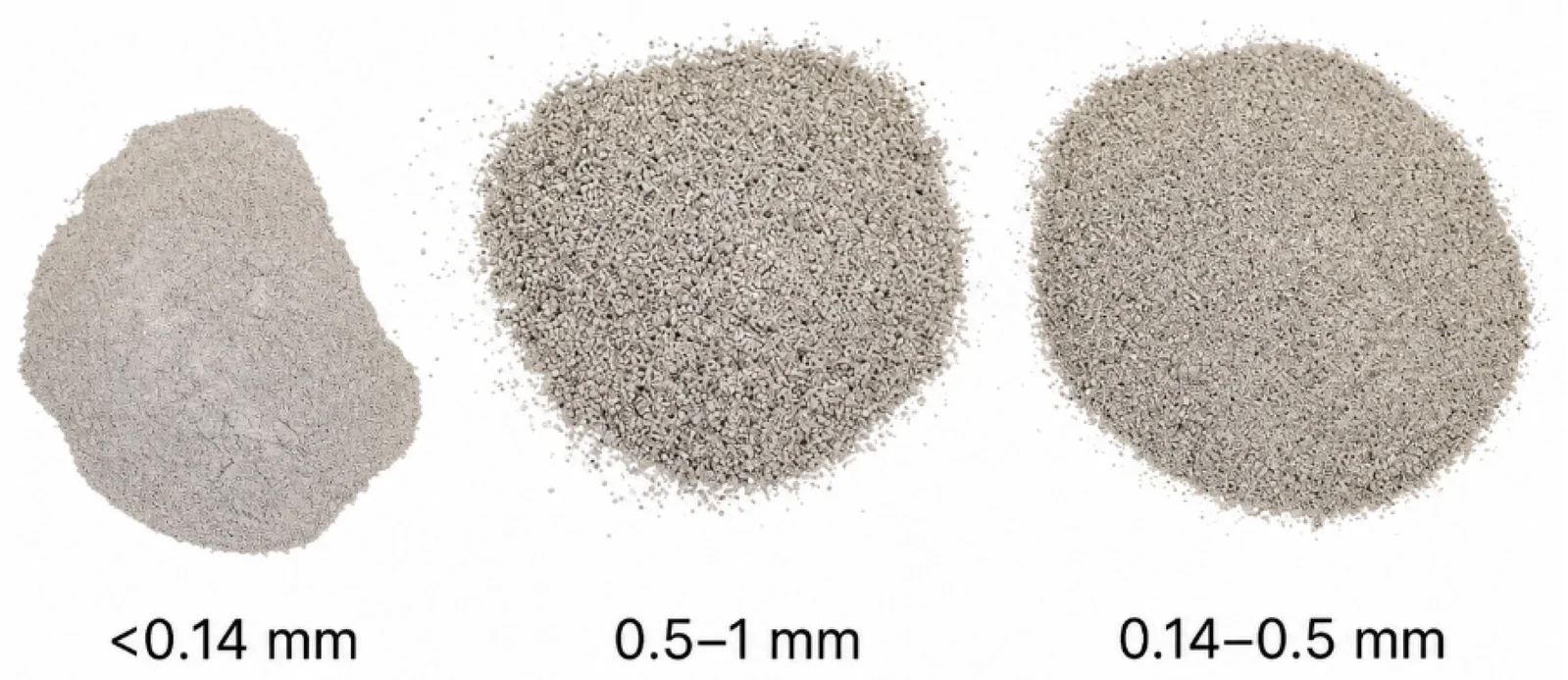
Fractions of concrete waste after crushing and sieving: <0.14 mm; 0.5–0.14 mm; 1–0.5 mm.

Recycled concrete powder (RCP) was obtained by crushing hardened concrete waste and separating its fine fractions. Two raw-material systems were investigated in two corresponding experimental series. Series I, hereafter referred to as the source-comparison series, included concrete waste prepared from concretes of different strength classes and ages, produced using granite crushed stone and quartz sand. After preliminary crushing, the material was processed in a jaw crusher; particles smaller than 1 mm were separated by sieving, and the <0.14 mm fraction was isolated as the fine RCP fraction for pressing. The obtained fractions are shown in Figure 1.

**Figure 2:**
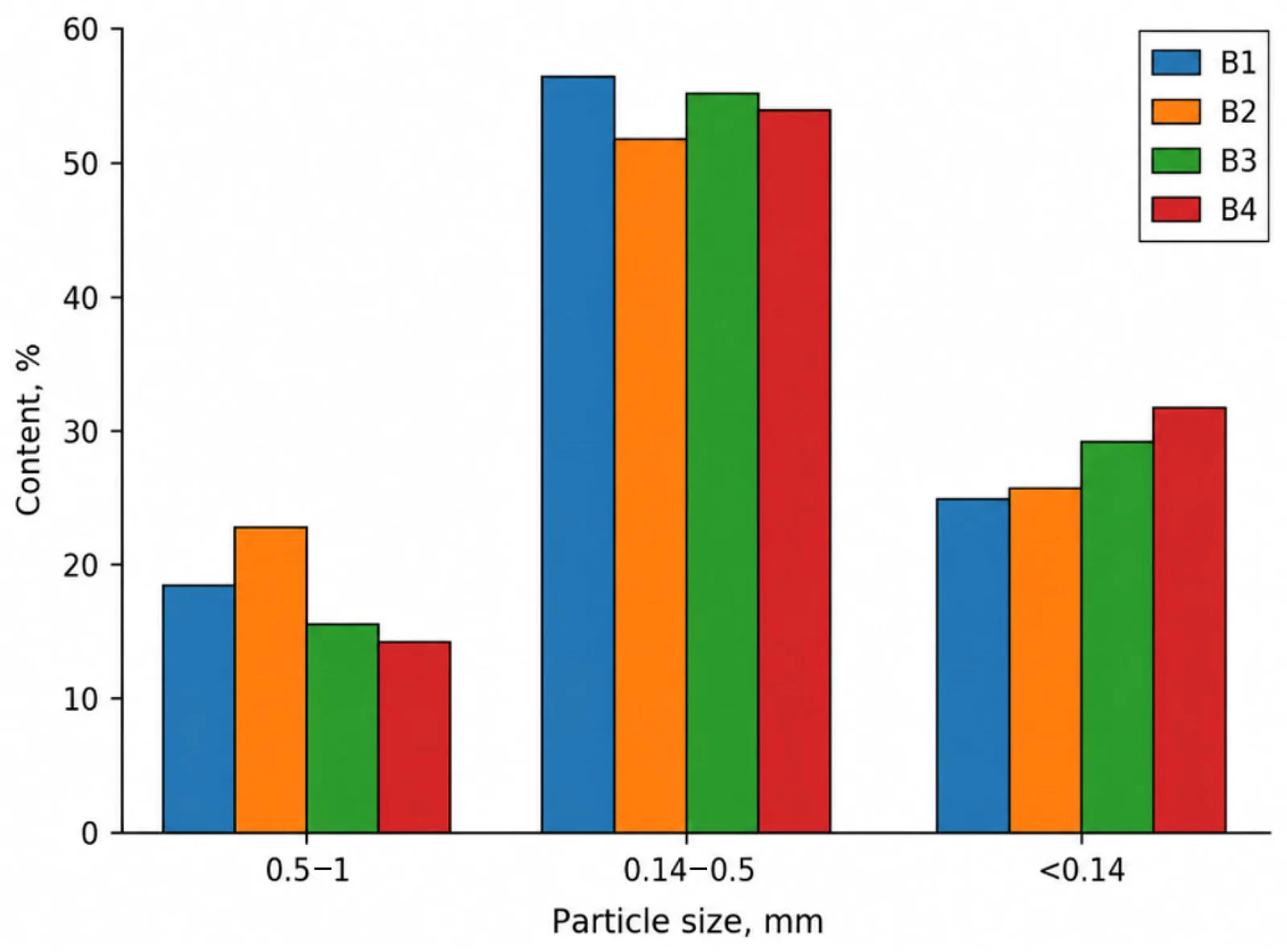
Sieve analysis data for concrete waste particles smaller than 1 mm.

The particle-size composition of the concrete waste fraction smaller than 1 mm was determined by sieve analysis (Figure 2). The <0.14 mm fraction was additionally characterized by laser diffraction using a Sympatec HELOS (H3960) particle-size analyzer (Sympatec GmbH, Clausthal-Zellerfeld, Germany) equipped with a RODOS dry dispersion unit and an R3 measuring range of 0.5/0.9–175 µm. The pH of aqueous extracts was measured as an indirect indicator of residual alkaline and calcium-containing phases. Within Series I, four RCP samples, denoted as B1–B4, were obtained from concretes differing in age, strength and residual cementitious potential (Table 1).

All four parent concretes (B1–B4) were processed using the same crushing and sieving sequence. No sample-specific crushing conditions or additional pretreatment procedures were applied. Therefore, the differences among B1–B4 discussed below are attributed primarily to the characteristics and history of the parent concretes rather than to differences in powder preparation.

The chemical and mineralogical composition of the fine fraction was determined by X-ray diffraction analysis using an Aeris diffractometer (Malvern Panalytical B.V., Almelo, The Netherlands), with attention to residual clinker minerals, calcium hydroxide, carbonate phases and quartz (Table 2).

The CaCO_3_ contents reported in Table 2 represent the semi-quantitatively determined contents of crystalline carbonate phases in the powders. They provide an indirect indication of carbonation-related mineralogical differences but should not be interpreted as direct measurements of the total carbonation degree. Dedicated quantitative carbonation tests were not included in the experimental programme.

**Figure 3:**
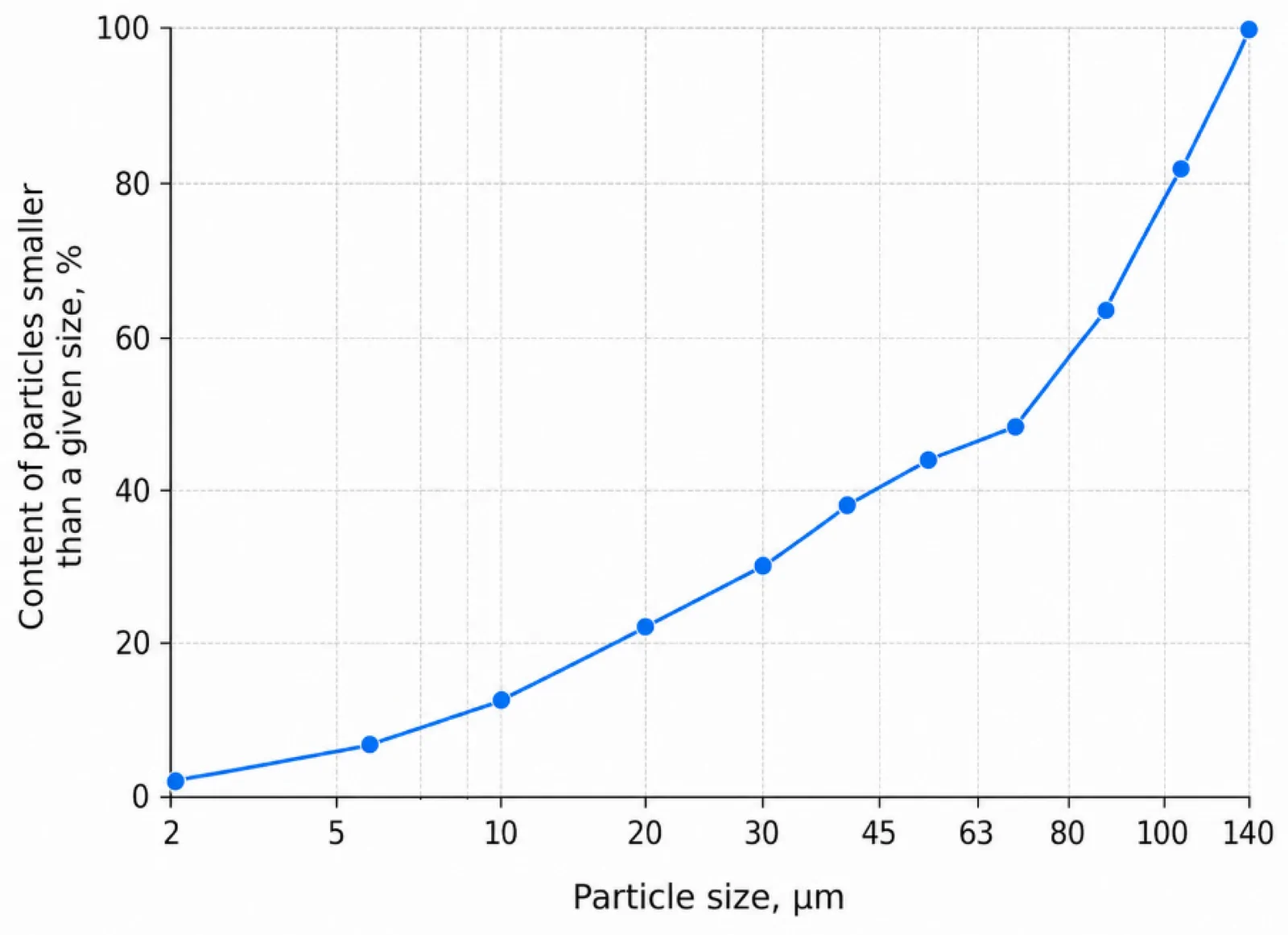
Particle-size distribution of the RCP fraction (<0.14 mm).

Series II, hereafter referred to as the processing-and-modification series, was based on concrete rubble collected from construction waste accumulation sites in the Kyiv region, Ukraine. The parent concrete was approximately 30–40 years old and corresponded roughly to strength class C20/25. After jaw crushing, the <1 mm fraction was separated and the <0.14 mm fraction was isolated for further use. Its particle-size distribution is shown in Figure 3, and its chemical and mineralogical composition is presented in Table 3. The designations B1–B4 are used exclusively for Series I. In the remainder of the manuscript, RCP, TRCP, RCP2.5, and RCP5 refer exclusively to materials derived from the Kyiv-region concrete waste used in Series II.

### 2.2. Pressed Material Compositions

Within Series II, four material systems were compared based on the <0.14 mm RCP fraction: untreated recycled concrete powder (RCP), thermally activated RCP (TRCP), RCP with 2.5 wt.% Portland cement (RCP2.5) and RCP with 5 wt.% Portland cement (RCP5). Thermal activation was carried out in a muffle furnace. The temperature was raised to 600 °C over 30 min and maintained at this temperature for 2 h. The thermally treated powder was then allowed to cool naturally in the closed furnace. For the low-cement systems, Portland cement was added to untreated RCP in amounts of 2.5 and 5 wt.% relative to the mass of RCP, followed by homogenization before moistening and pressing. Portland cement CEM I 42.5 (VIPCEM, Kyiv, Ukraine) with the following chemical composition (%) was used: SiO_2_—22.47, Al_2_O_3_—5.26, Fe_2_O_3_—4.07, CaO—66.18, MgO—0.64, SO_3_—0.46, and MnO—0.29.

### 2.3. Specimen Preparation and Curing

**Figure 4:**
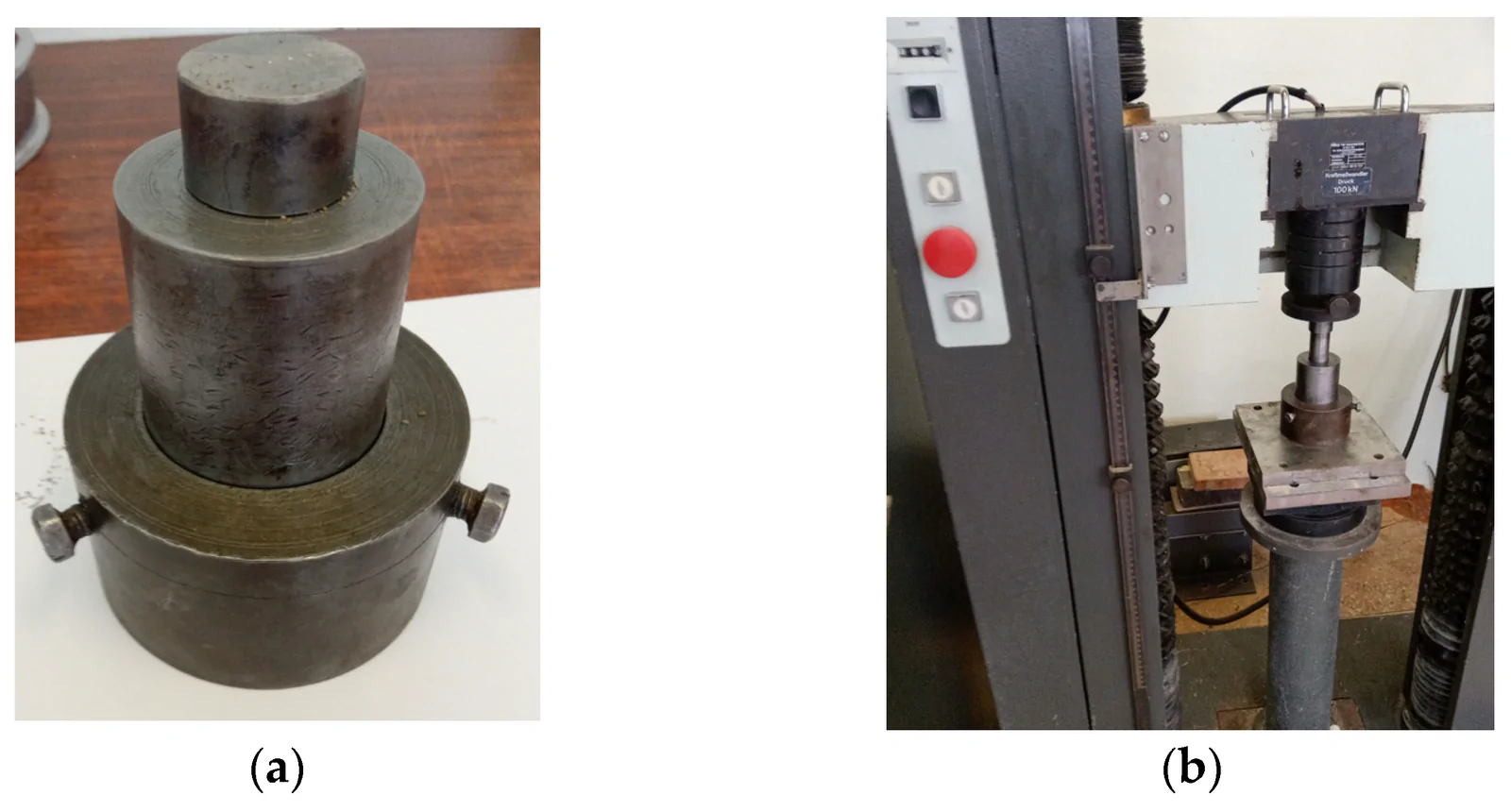
Equipment for forming pressed specimens: (**a**) mold for cylindrical specimens; (**b**) specimen forming using the FP 100/1 testing machine.

Pressed specimens were produced by semi-dry pressing. Cylindrical specimens with a diameter and height of 25 mm were formed in a steel mold using an FP 100/1 testing machine (VEB Fritz-Heckert-Werk, Chemnitz, Germany). For the principal Series II tests, the forming moisture content was 12–13%, and the pressing pressure was 20 MPa. The equipment used for specimen preparation is shown in Figure 4.

**Figure 5:**
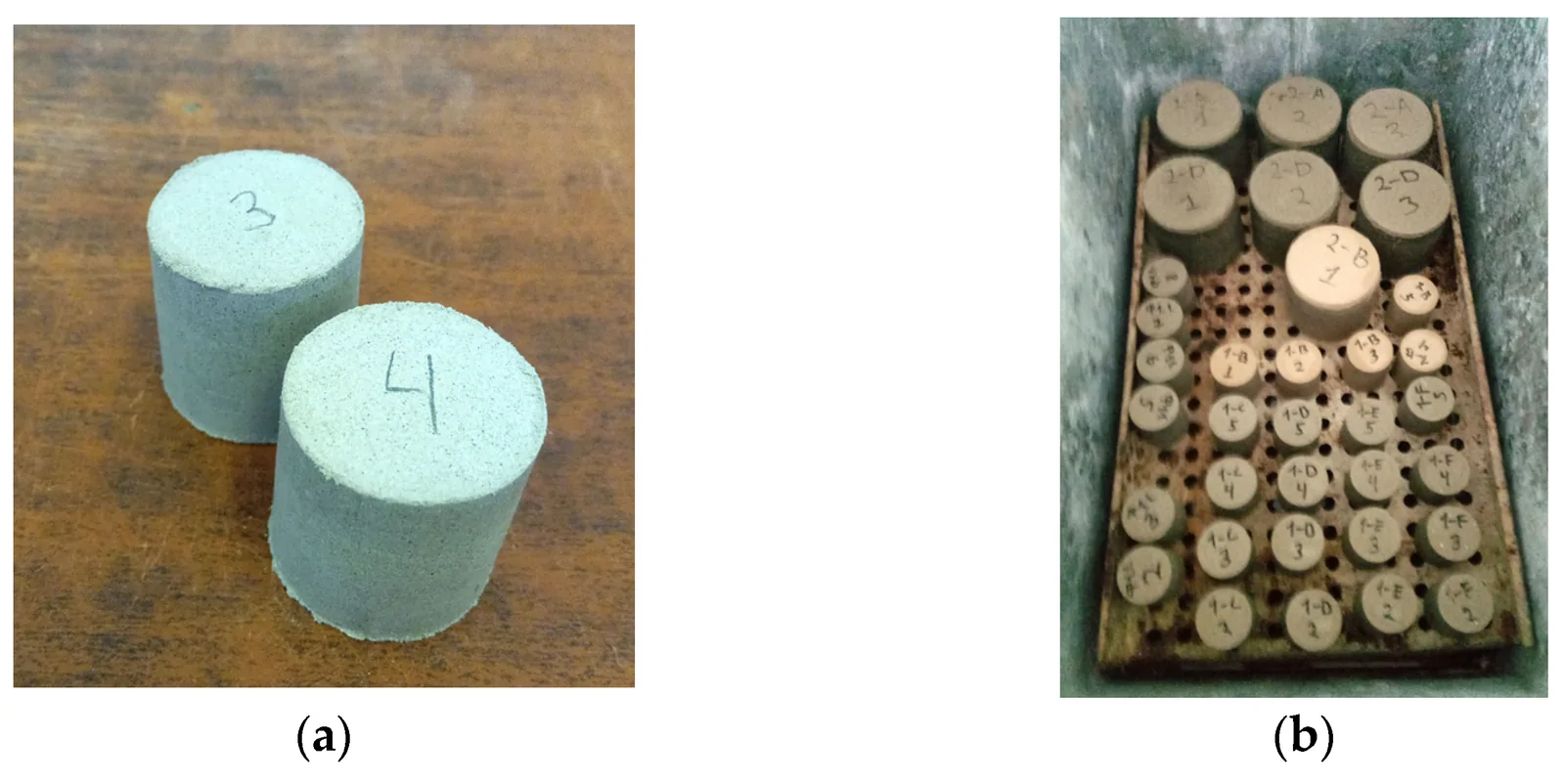
Pressed specimens based on recycled concrete powder and curing conditions: (**a**) cylindrical specimens after forming; (**b**) storage of specimens in a desiccator above water. The handwritten numbers and letters are laboratory specimen identification codes.

Three curing regimes were compared: air-dry curing, humid-air curing in a desiccator above water and water curing after 1 day of preliminary air curing. The main Series II comparative tests were performed on specimens cured under humid-air conditions at 20 ± 2 °C and relative humidity close to 100% (Figure 5). For each composition and curing age, three specimens were tested. The results were statistically processed by calculating the arithmetic mean, sample standard deviation, and coefficient of variation. Unless otherwise stated, the experimental values are presented as the mean ± standard deviation (*n* = 3). The coefficients of variation did not exceed 6.9% for compressive strength, 2.0% for bulk density, and 4.4% for the water resistance coefficient, indicating limited within-series variability under the adopted specimen preparation and testing conditions.

### 2.4. Testing Methods

**Figure 6:**
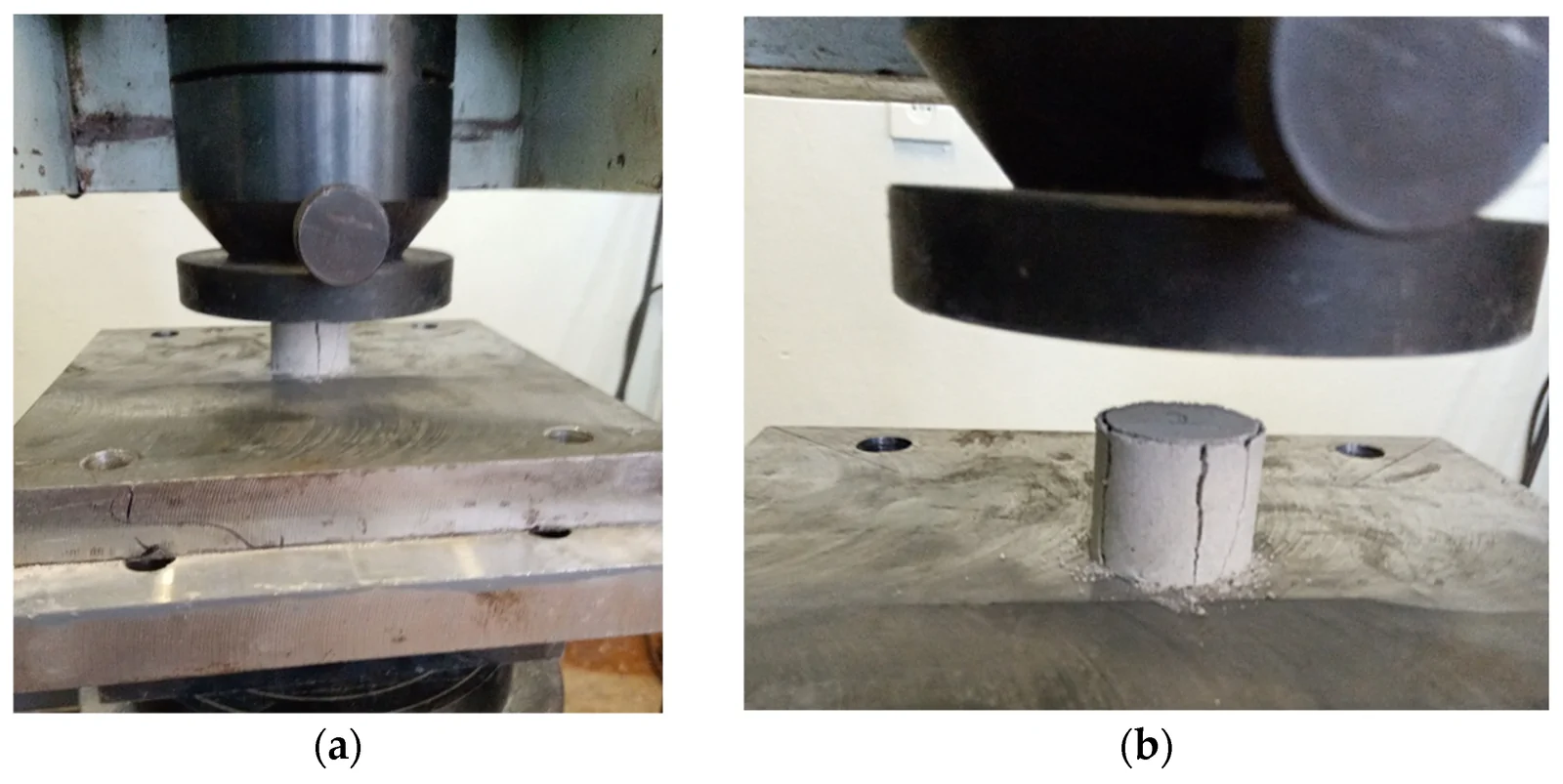
Compressive testing of pressed specimens: (**a**) specimen placed between the press plates; (**b**) typical failure pattern after loading.

The pressed materials were characterized by compressive strength, bulk density and water resistance coefficient. For Series I samples B1–B4, compressive strength was determined at 1 h, 1 day, 7 days and 28 days. For the Series II systems RCP, TRCP, RCP2.5 and RCP5, the main tests were performed after 3, 7 and 28 days of curing. Compressive testing and the typical failure pattern are shown in Figure 6.

The water resistance coefficient was calculated as the ratio between the compressive strength of specimens saturated in water for 24 h and that of corresponding dry specimens. This parameter was used to characterize the stability of the pressed structure under water action.

Within Series II, the experimental programme comprised two stages. The first stage was a technological screening intended to identify suitable forming moisture, pressing pressure, and curing conditions. The effects of moisture content and pressing pressure were evaluated primarily using 7-day compressive strength, whereas the curing-regime experiment followed strength development up to 54 days. After selecting the forming conditions, the principal comparative stage was performed for RCP, TRCP, RCP2.5, and RCP5, for which compressive strength, bulk density, and water resistance coefficient were determined at 3, 7, and 28 days. Water resistance was not determined separately for every condition included in the preliminary screening stage.

### 2.5. Thermal Analysis

For Series II materials, differential thermal and thermogravimetric analyses were performed using a Shimadzu DTG-60 instrument (Shimadzu Corporation, Kyoto, Japan) in air. The temperature range was 50–800 °C, and the heating rate was 20 °C/min. Untreated RCP, thermally activated RCP and hardened pressed specimens based on RCP, TRCP, RCP2.5 and RCP5 were analyzed. The DTA/TGA data were used to evaluate thermal effects and mass losses associated with physically bound water, hydrated phases and carbonate components.

### 2.6. Experimental Design and Statistical Analysis of Multifraction Compositions

Within Series II, a separate multifraction experiment was carried out using fractions obtained from the same Kyiv-region concrete-waste raw-material system to evaluate the effect of multifraction composition on pressed composites. A three-factor three-level B_3_ experimental design was used [36]. The independent variables were the content of the <0.14 mm fraction, the 0.14–0.5 mm fraction and Portland cement. The contents of the three RCP fractions were expressed as percentages of the total RCP mass and summed to 100%. Portland cement was introduced separately in an amount of 0–5 wt.% relative to the RCP mass. The 0.5–1 mm fraction was calculated as the remaining part of the concrete waste fraction system. The response parameters were the 28-day compressive strength and the water resistance coefficient.

All specimens in this series were formed at 20 MPa with a forming moisture content of 12% and cured under humid-air conditions at a relative humidity of 95–100%. The data were processed using full quadratic regression models in coded variables to assess the effects of the factors and identify a rational composition range.

Model performance was evaluated using the coefficient of determination (R^2^), adjusted coefficient of determination (adjusted R^2^), root mean square error (RMSE), mean absolute error (MAE), and the range of residuals calculated as the differences between the experimental and fitted values. Because all 17 experimental design points were used to estimate the model coefficients, no independent validation dataset was available. The models were therefore intended for interpolation and comparative analysis within the investigated factor ranges rather than for extrapolation beyond the experimental domain.

## 3. Results and Discussion

### 3.1. Effect of the Origin and Composition of Recycled Concrete Powder on the Properties of Pressed Materials

This subsection presents the results of Series I, which was designed to evaluate the influence of the parent concrete source and residual mineralogical state. The properties of pressed materials based on RCP are influenced not only by pressing parameters but also by the origin of the parent concrete. To evaluate this effect, four fine powder samples, B1–B4, were pressed from the <0.14 mm fraction under the same pressure of 20 MPa. Their source characteristics and mineralogical compositions were given in Table 1 and Table 2; therefore, differences in the properties of the pressed specimens can mainly be related to the residual reactivity of the powders.

The test results are summarized in Table 4.

At early ages, all specimens had similar strength: 2.9–3.2 MPa after 1 h and 3.5–4.6 MPa after 1 day. This indicates that the initial strength was governed mainly by mechanical compaction, capillary forces, interparticle friction and primary adhesion. At later ages, the differences became much more pronounced: after 28 days, strength ranged from 5.6 MPa for B4 to 13.4 MPa for B1. The materials can be arranged by 28-day strength as B1 > B3 > B2 > B4.

This order is not controlled only by the nominal strength of the parent concrete. B2 originated from high-strength C40/50 concrete but exhibited lower pressed strength than B3, which was obtained from lower-strength C16/20 concrete. The decisive factor is therefore the state of the cementitious component after crushing. B1 contained the highest residual C_3_S content (14.22%), a relatively high Ca(OH)_2_ content (9.03%) and the highest pH (11.7), which indicates preserved residual hydraulic potential. By contrast, B4 had almost no residual C_3_S (0.22%), the lowest Ca(OH)_2_ content (2.41%) and the lowest pH (8.9), while its high SiO_2_ content indicated the predominance of relatively inert aggregate particles.

The higher 28-day strength of B1 should therefore be interpreted as being consistent with a greater residual hydraulic potential rather than as proof of the action of any single phase. Residual C_3_S can undergo further hydration after crushing exposes new particle surfaces, while the alkaline environment associated with Ca(OH)_2_ facilitates the dissolution and transport of calcium- and silicate-bearing species. Their subsequent precipitation in the restricted interparticle space may strengthen the contact zones. Conversely, the substantially lower residual C_3_S and Ca(OH)_2_ contents and the lower pH of B4 are consistent with its weaker strength development. Because these mineralogical characteristics vary simultaneously with the origin of the powder, their individual contributions cannot be quantitatively separated using the present data.

The CaCO_3_ content alone did not determine the performance ranking of the powders. B1, which contained 1.59% CaCO_3_, showed the highest 28-day compressive strength (13.4 MPa) and water resistance coefficient (0.92), whereas B2 had the highest CaCO_3_ content (6.63%) but reached only 8.7 MPa and a water resistance coefficient of 0.87. B3, with 2.39% CaCO_3_, developed higher strength and water resistance than B2, while B4 combined a relatively high CaCO_3_ content (4.89%) with the lowest residual C_3_S and Ca(OH)_2_ contents, the lowest pH, and the lowest 28-day strength. These comparisons indicate that the properties were governed by the combined mineralogical state and alkalinity of the powders rather than by carbonate content alone. Because only four powder sources were examined and their characteristics varied simultaneously, these relationships should be regarded as comparative trends rather than statistically independent correlations.

**Figure 7:**
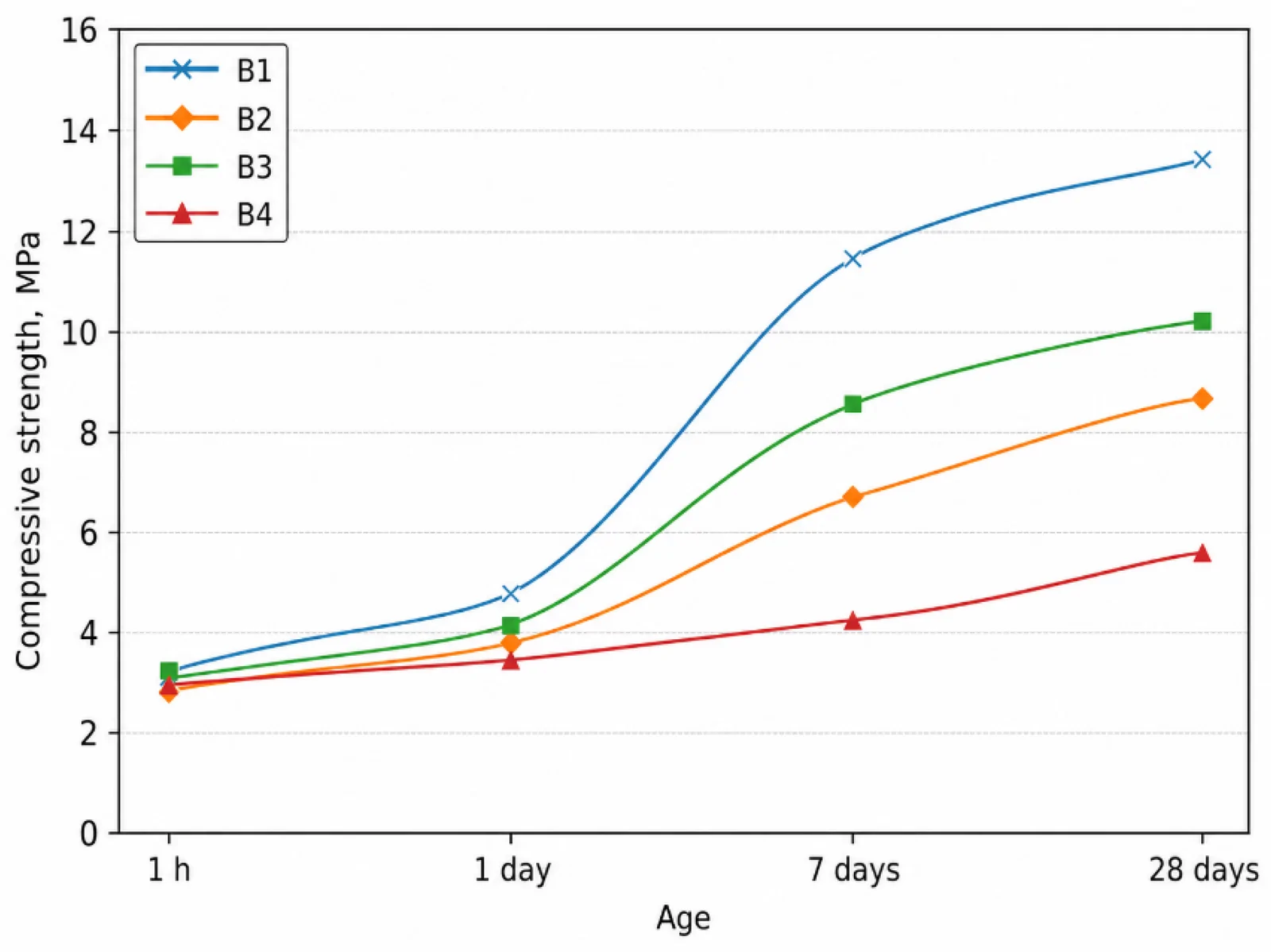
Strength development of pressed specimens over time.

The strength development of the pressed specimens is shown in Figure 7.

The curves confirm that all RCP-based pressed materials gained strength with curing time, which means that they should not be regarded as only mechanically compacted powders. The most intensive strength gain occurred between 1 and 7 days. The differences in compressive strength were much greater than the differences in dry density, which varied only from 1.56 to 1.62 g/cm^3^. Therefore, strength was controlled not only by densification but also by the quality of interparticle contacts, the development of new binding products and the mineralogical composition of the powder.

The 28-day water resistance coefficient was relatively high for all sources, ranging from 0.87 to 0.92. Thus, the structure formed during pressing and humid-air curing was sufficiently stable under water saturation, although the absolute strength level depended strongly on the residual cementitious potential of RCP. The contents of C_3_S and Ca(OH)_2_, together with pH, can therefore be used as preliminary indicators of RCP suitability, but a full assessment should also include carbonation degree, particle-size distribution and the amount of old cement paste.

### 3.2. Effect of Technological Parameters on the Formation of Pressed Materials

Series II was used to investigate the effects of forming moisture content, pressing pressure, and curing regime. The properties of pressed RCP-based materials are determined by forming moisture content, pressing pressure and curing regime. Water has a dual role: it facilitates particle rearrangement during pressing and provides the medium for residual hydration and dissolution–precipitation processes after pressing. Excess water, however, can weaken contacts and generate additional porosity.

This subsection presents the preliminary technological-screening results; it should therefore be distinguished from the principal material comparison reported in Section 3.3.

**Figure 8:**
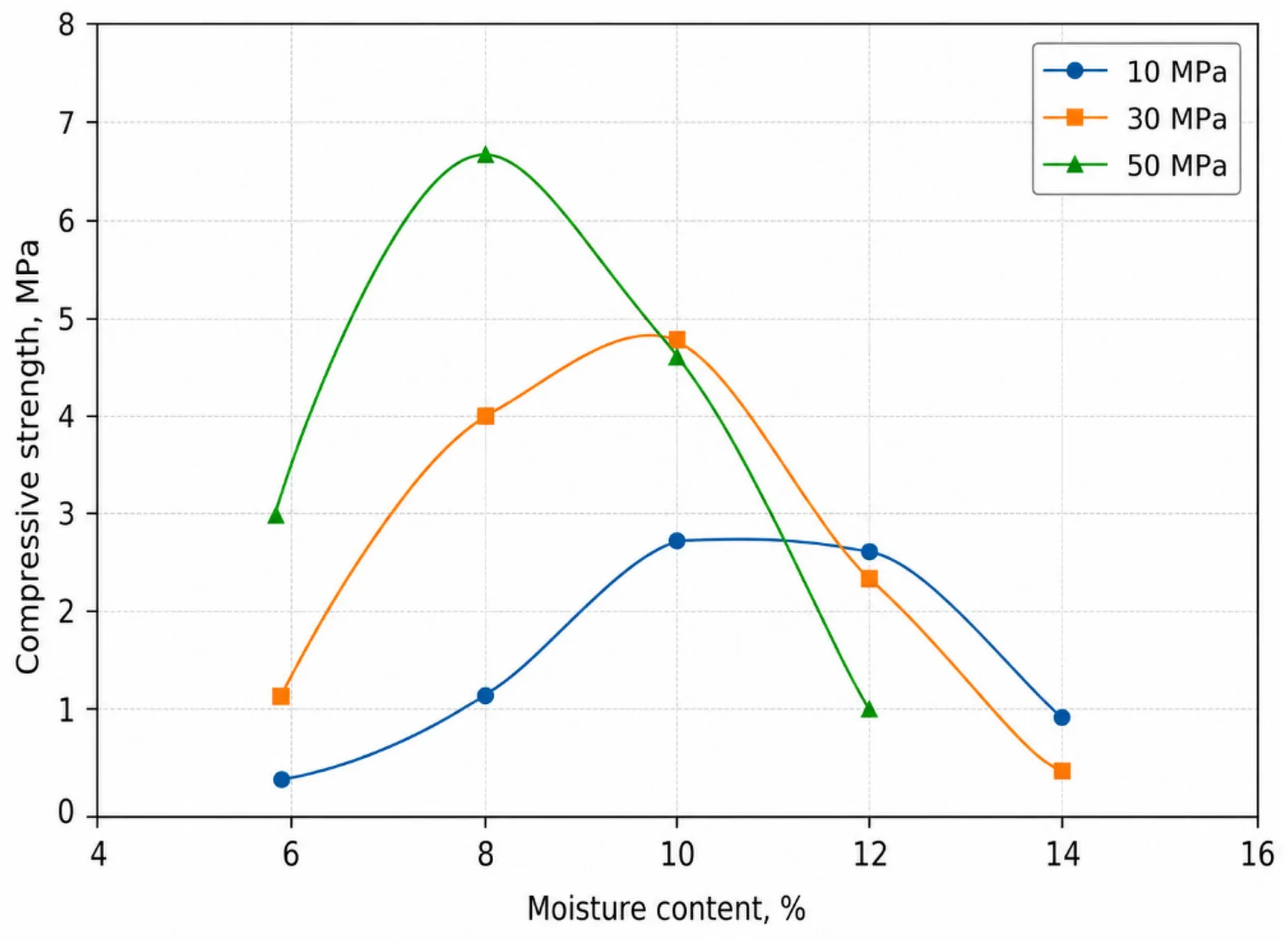
Effect of forming moisture content of the powder on the compressive strength of pressed composites.

The effect of moisture content at different pressing pressures is shown in Figure 8.

For each pressing pressure, the 7-day strength exhibited a non-monotonic dependence on moisture content. At 10 MPa, the optimum was approximately 10–12%; at 30 MPa, it shifted to about 10%; and at 50 MPa, to about 8%. Higher pressure partly compensated for lower moisture by more intensive particle rearrangement and denser packing. The decrease in strength at excessive moisture is attributed to the presence of an excess liquid phase, which prevents stable contact formation and leaves pores after drying or redistribution.

**Figure 9:**
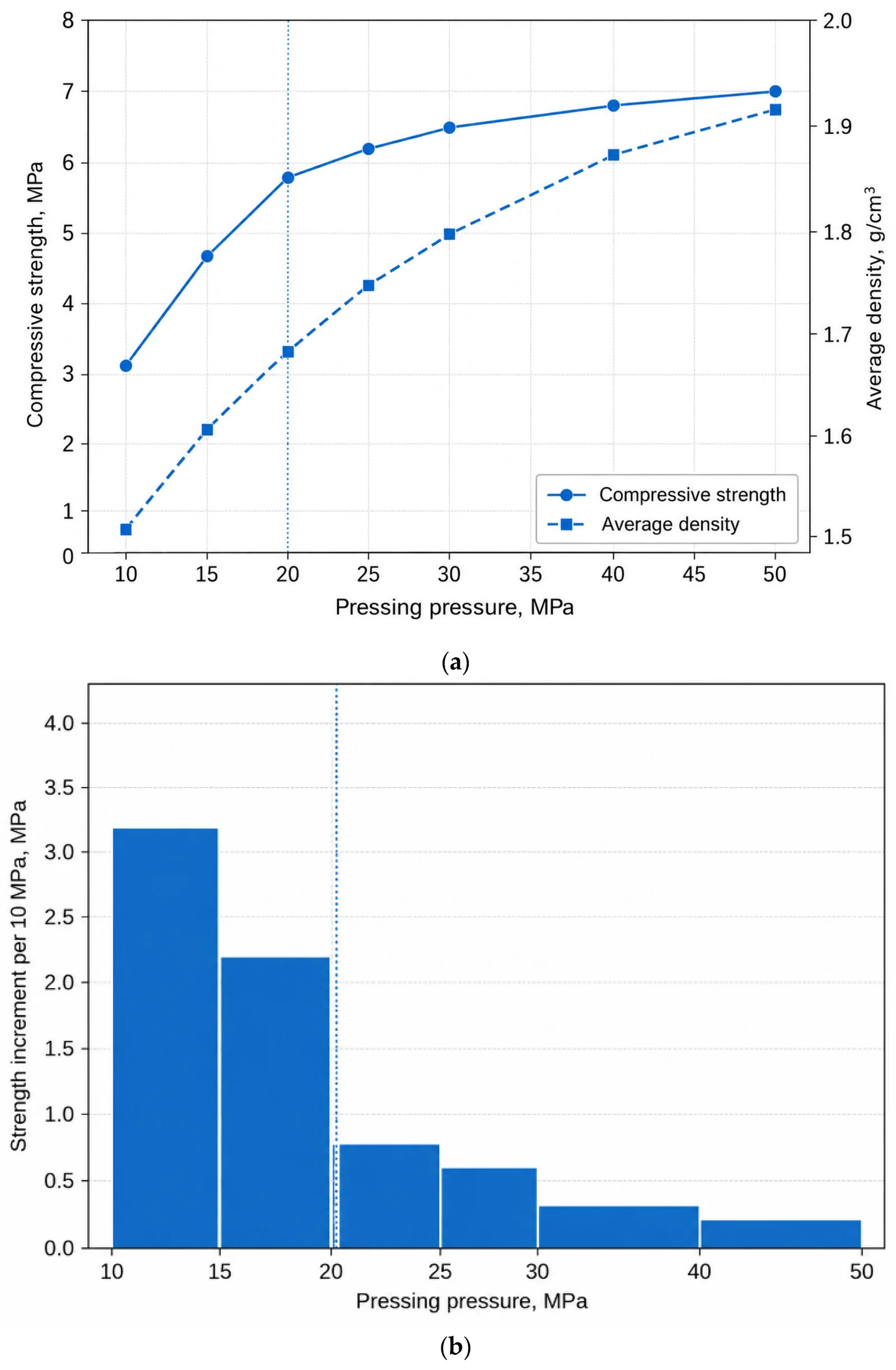
Effect of pressing pressure on the properties of pressed materials based on RCP: (**a**) dependence of compressive strength and bulk density on pressing pressure; (**b**) change in strength increment with increasing pressing pressure. The vertical dashed line indicates the selected rational pressing pressure of 20 MPa.

The effect of pressing pressure on strength and bulk density is presented in Figure 9.

Increasing the pressure from 10 to 50 MPa increased both density and compressive strength, confirming the role of mechanical compaction. However, the pressure effect was nonlinear: after approximately 20 MPa, the additional strength increment decreased. From a technological perspective, the maximum pressure is therefore not necessarily rational. For the investigated system, 20 MPa was selected as a balanced level that ensured sufficient compaction without excessive equipment loading.

**Figure 10:**
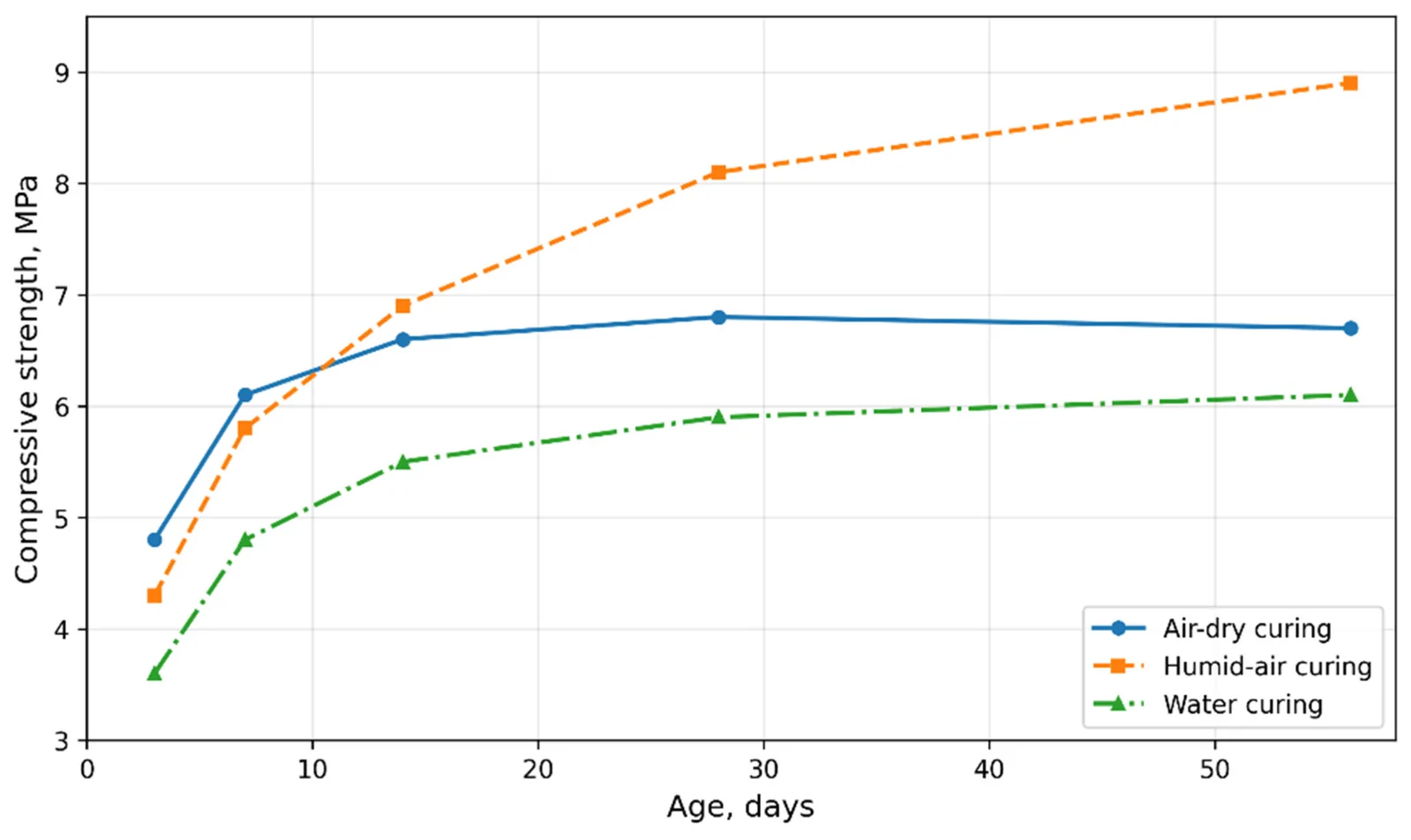
Effect of curing conditions on the compressive strength of pressed materials based on RCP.

The effect of curing conditions is shown in Figure 10.

At 28 days, the compressive strengths under air-dry, humid-air, and water curing were approximately 6.8, 8.1, and 5.9 MPa, respectively. Thus, the superiority of humid-air curing was maintained beyond the early testing ages.

Air-dry curing limited further hardening because of rapid moisture loss. Water curing was also not optimal, probably because excessive water saturation disturbed the stability of contact zones. The best results were obtained under humid-air curing above water at relative humidity close to 100%, where specimens retained moisture without being fully saturated. Therefore, the rational technological combination adopted for the main series was a forming moisture content of 12–13%, a pressure of 20 MPa and humid-air curing.

The advantage of humid-air curing further supports this interpretation. Under these conditions, thin water films remain available at particle contacts for residual hydration and dissolution–precipitation processes, while the specimens are not completely water-saturated. The lower strength under air-dry curing is consistent with the premature limitation of these aqueous processes. The lower performance under water curing may reflect a reduced contribution of capillary forces and lower stability of the early contact zones; however, the present experiments do not allow these effects to be separated quantitatively.

The screening stage was intended to select processing conditions rather than to generate a complete 28-day property matrix for every moisture–pressure combination. Consequently, 28-day strength and water resistance were not determined for all screening conditions. The selected conditions were subsequently evaluated in greater detail in the principal comparative series presented in Section 3.3.

### 3.3. Properties of Cement-Free, Thermally Activated and Low-Cement Pressed Systems

Within Series II, after selecting rational forming and curing parameters, four systems were compared: untreated RCP, thermally activated RCP (TRCP), RCP with 2.5 wt.% Portland cement (RCP2.5) and RCP with 5 wt.% Portland cement (RCP5). All specimens were formed at 20 MPa with 12–13% moisture and cured under humid-air conditions. The results are presented in Table 5.

Untreated RCP formed a cement-free pressed material with gradual strength development from 4.3 MPa at 3 days to 6.9 MPa at 28 days. This increase indicates that hardening continued after pressing, although the residual reactivity of the untreated powder was limited. Thermal activation increased the 28-day strength to 11.4 MPa, approximately 1.65 times that of untreated RCP. Heating at 600 °C can partially dehydrate calcium silicate hydrates and dehydroxylate calcium hydroxide in the old cement paste, producing transformed calcium- and silicate-bearing phases with the potential for renewed interaction with water. During subsequent humid-air curing, rehydration and dissolution–precipitation processes may therefore contribute to the strengthening of interparticle contact zones. In the present study, this interpretation is indirectly supported by the decreased mass loss of the activated powder in the 105–500 °C range and by its pronounced later-age strength gain. Kim and Ubysz [22] likewise reported that thermal treatment at 600 and 800 °C improved the strength and shrinkage resistance of mortars containing recycled concrete powder. Hashmi et al. [24], however, identified 400 °C as the most efficient thermal treatment in a combined thermal–carbonation activation system, indicating that the optimum activation temperature depends on the origin of RCP, the subsequent activation route, and the material in which it is used. Unlike these studies, where RCP was introduced as a partial cement replacement in mortar, the present system uses it as the principal mineral matrix of the pressed composite. Since no direct imaging or pore-structure analysis was performed, the proposed modification of the contact zones should be regarded as a mechanism consistent with the strength and thermoanalytical results rather than as directly observed microstructural evidence.

Portland cement additions mainly increased early strength and water resistance. RCP2.5 reached 10.6 MPa after 28 days, while RCP5 reached 12.8 MPa and showed the highest bulk density (1.76 g/cm^3^) and water resistance coefficient (0.86). Even a very small amount of fresh cement therefore produced hydration products in the limited pore space and efficiently strengthened interparticle contact zones.

**Figure 11:**
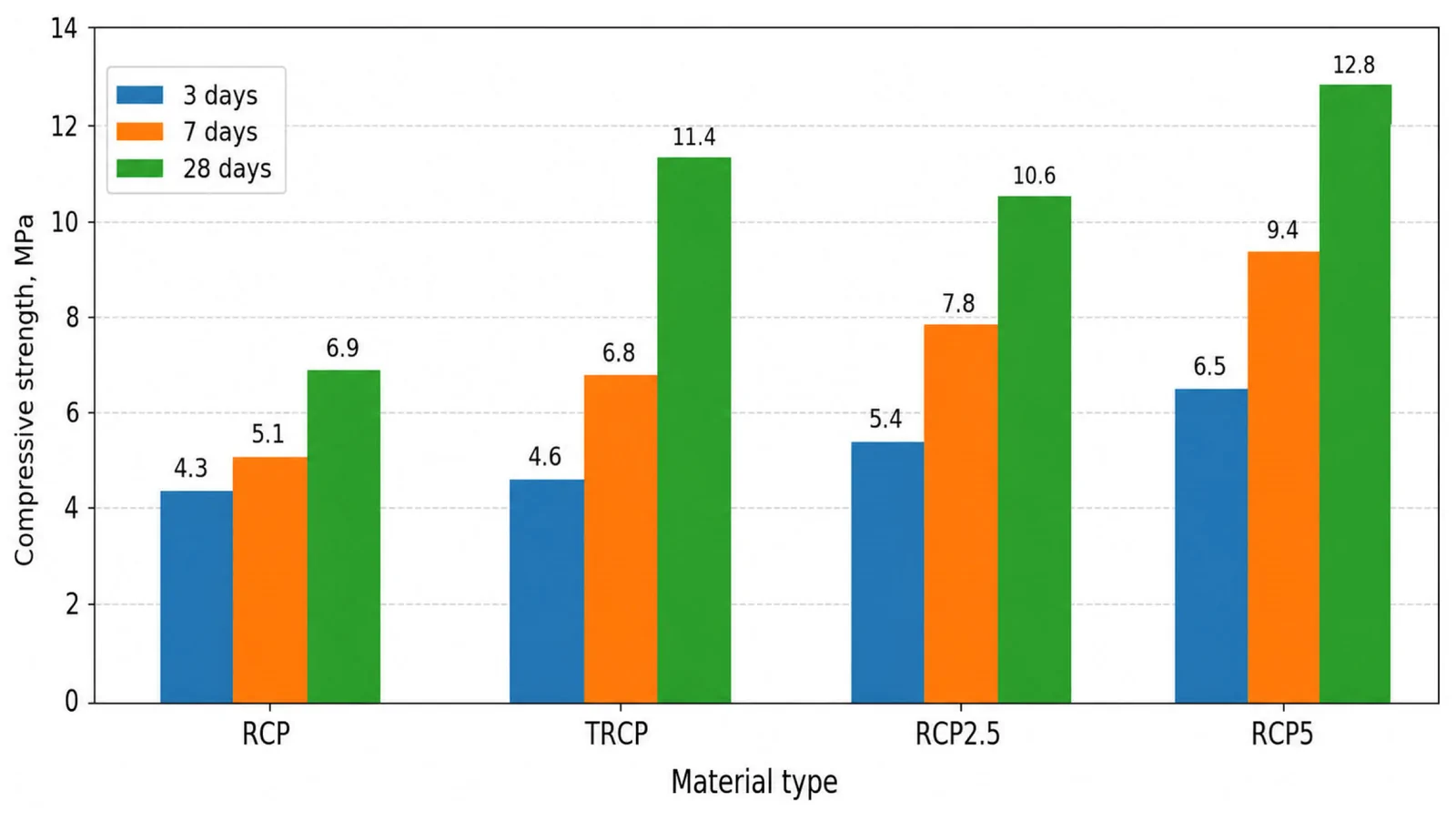
Compressive strength of RCP-based pressed materials at different curing ages.

The strength development of the investigated systems is shown in Figure 11.

All systems gained strength over time, but the intensity differed. Untreated RCP had the lowest rate of increase, TRCP showed a pronounced later-age gain, and the low-cement systems combined higher early strength with stable further hardening. Density differences were smaller than strength differences, which confirms that strength improvement was governed not only by physical densification but also by activation, contact hardening and the formation of additional binding products.

Water resistance increased in the order RCP < TRCP < RCP2.5 < RCP5. For untreated RCP, the 28-day coefficient was 0.61; thermal activation increased it to 0.74, while 2.5 and 5 wt.% cement increased it to 0.80 and 0.86, respectively. Thus, thermal activation is effective for improving strength without additional clinker, whereas small cement additions are particularly effective for stabilizing the structure under water action.

### 3.4. Multifraction Composition Design and Regression Modelling

For the multifraction experiment within Series II, the preceding results indicated that the use of only one fine fraction does not fully exploit concrete waste as a multifraction mineral system. Therefore, a B_3_ experimental design [36] was used to evaluate the combined effect of particle-size composition and Portland cement content on 28-day compressive strength and water resistance. All specimens were pressed at 20 MPa, with 12% moisture, and cured under humid-air conditions. The factor levels are given in Table 6.

The content of the 0.5–1 mm fraction was calculated from the material balance C_0_._5–1_ = 100 − X_1_ − X_2_. The transition to coded variables was performed as x_1_ = (X_1_ − 35)/15, x_2_ = (X_2_ − 25)/10 and x_3_ = (X_3_ − 2.5)/2.5. The experimental matrix and measured properties are given in Table 7.

The 28-day strength varied from 5.4 to 11.5 MPa, and the water resistance coefficient varied from 0.56 to 0.84. The lowest values were obtained for cement-free compositions with unfavorable fractional combinations, whereas the highest values corresponded to compositions with 5% cement and increased contents of fine and medium fractions. Statistical processing of the experimental data yielded quadratic regression equations that can be regarded as experimental–statistical models describing the effects of the investigated factors on the response parameters. The resulting models and their coefficients of determination are presented in Table 8.

The coefficients of determination indicate that the models explain 96.0% and 95.1% of the experimental variation in compressive strength and water resistance coefficient, respectively. The corresponding adjusted R^2^ values were 0.908 and 0.888. Residual analysis yielded RMSE values of 0.35 MPa for compressive strength and 0.018 for the water resistance coefficient, while the respective MAE values were 0.30 MPa and 0.016. The residuals ranged from −0.56 to +0.54 MPa for compressive strength and from −0.027 to +0.029 for the water resistance coefficient and were centered around zero. These results indicate a reasonably close fit to the experimental design data. However, they represent internal goodness-of-fit diagnostics rather than independent model validation. Because the 17-point design was used to estimate ten coefficients in each full quadratic model and no additional validation compositions were tested, the predictive robustness of the models outside the investigated factor ranges has not been established.

In the strength model, the strongest positive effect was produced by Portland cement content, followed by the <0.14 mm fine fraction. Cement provides hydration products that bind particles in contact zones, while the fine fraction increases packing density, fills microvoids and contains the largest proportion of old cement paste residues. The medium fraction also had a positive but smaller effect related mainly to packing optimization. Negative quadratic coefficients indicate that the composition should be balanced rather than based on maximizing a single component.

**Figure 12:**
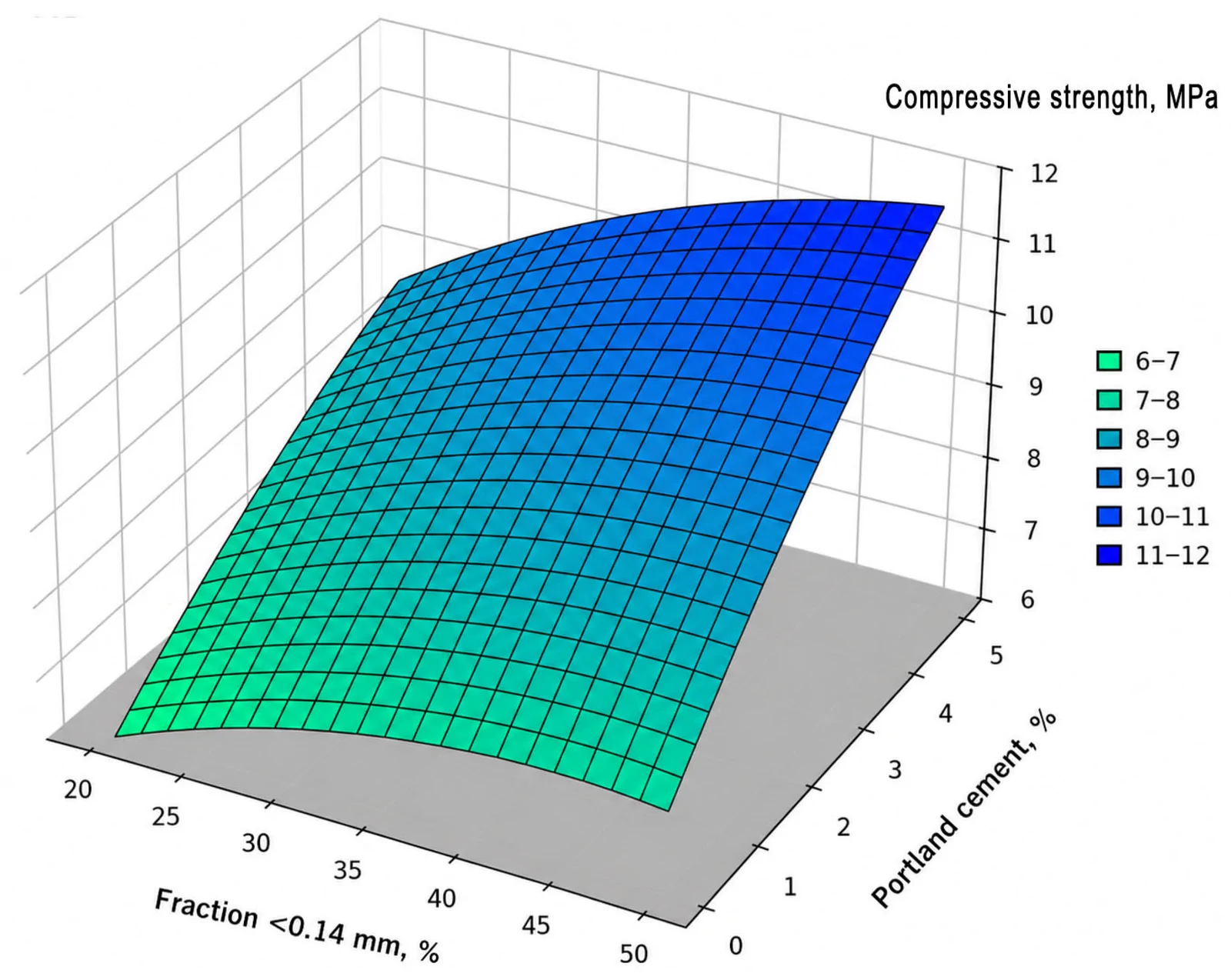
Response surface of 28-day compressive strength as a function of the <0.14 mm fraction content and Portland cement content at a fixed 0.14–0.5 mm fraction content of 25% (x_2_ = 0).

The response surface for compressive strength is shown in Figure 12.

**Figure 13:**
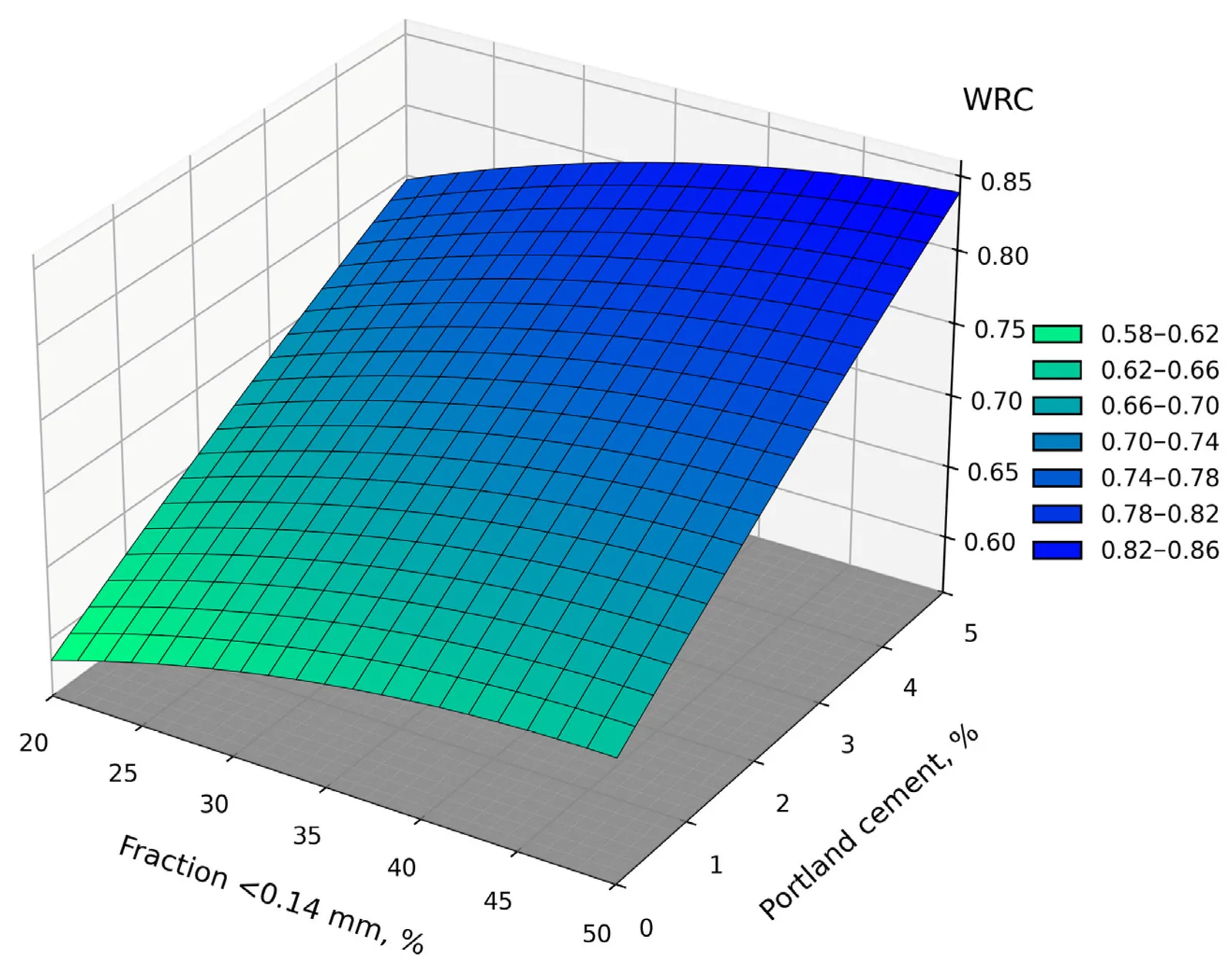
Response surface of the water resistance coefficient as a function of the <0.14 mm fraction content and Portland cement content at a fixed 0.14–0.5 mm fraction content of 25% (x_2_ = 0).

The surface shows that strength increases mainly with cement content and also with the fine fraction content, although the latter effect becomes less intense at high contents. For water resistance, Portland cement again had the strongest positive effect, while the fine and medium fractions improved contact-zone densification and packing. The corresponding response surface is shown in Figure 13.

**Figure 14:**
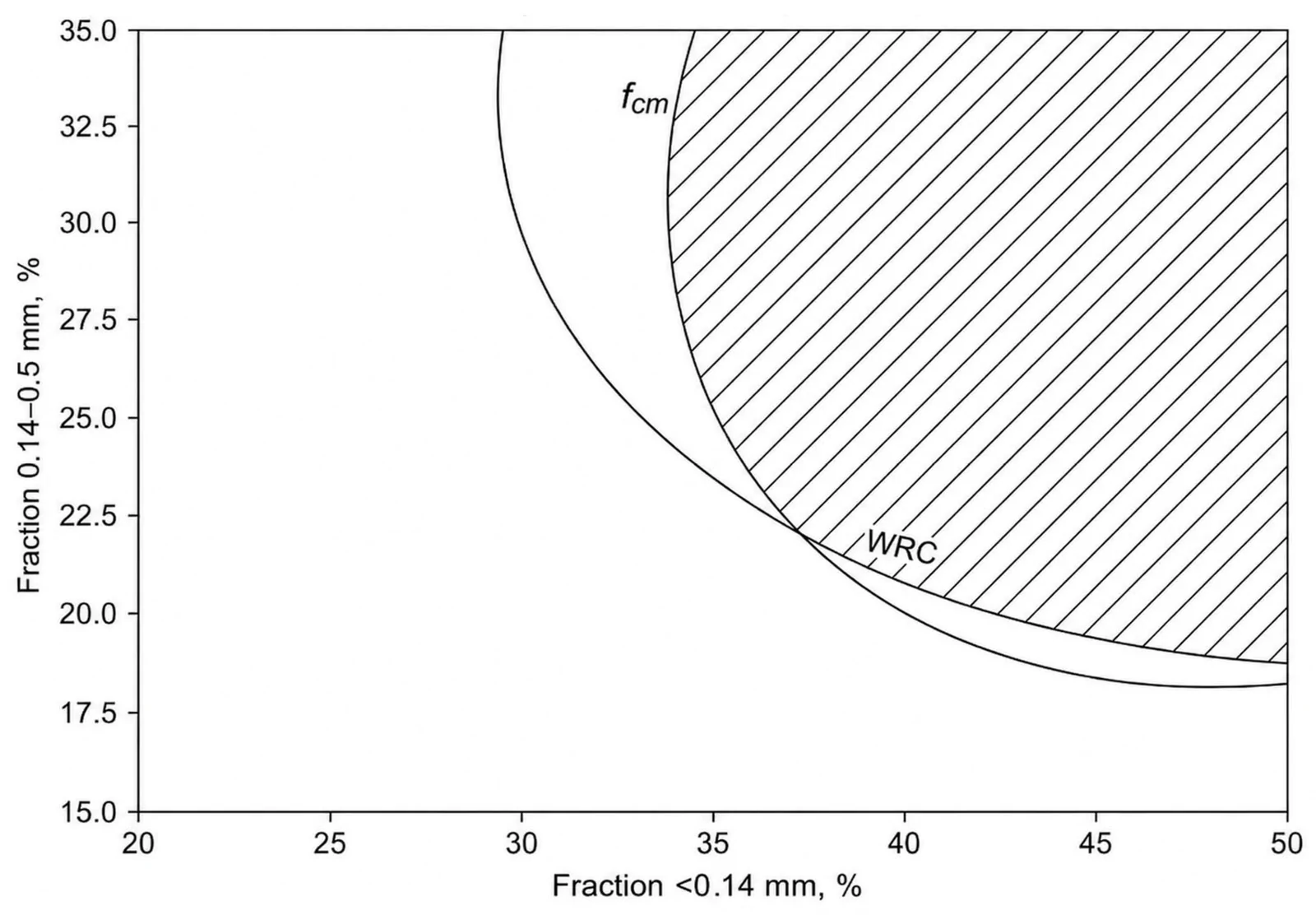
Generalized diagram of the rational composition region at 2.5% cement content under the conditions f_cm_ ≥ 9.0 MPa and WRC ≥ 0.72.

To delineate an exploratory rational composition range, both models were analyzed jointly at 2.5% cement content using the criteria f_cm_ ≥ 9.0 MPa and WRC ≥ 0.72. The resulting model-based region is shown in Figure 14.

The rational region corresponds approximately to 36–50% of the <0.14 mm fraction, 22–35% of the 0.14–0.5 mm fraction and 15–42% of the 0.5–1 mm fraction. Thus, the properties of pressed composites are governed not only by cement content but also by a rational combination of fractions: the fine fraction fills microvoids and contributes residual activity, the medium fraction improves packing, and excessive coarse fraction content reduces contact-zone activity.

Accordingly, the proposed composition range should be regarded as a model-based estimate within the investigated experimental domain. Independent experiments with compositions selected within and near the boundaries of this region are required for its final validation.

### 3.5. Thermoanalytical Evidence of Phase Evolution During Hardening

**Figure 15:**
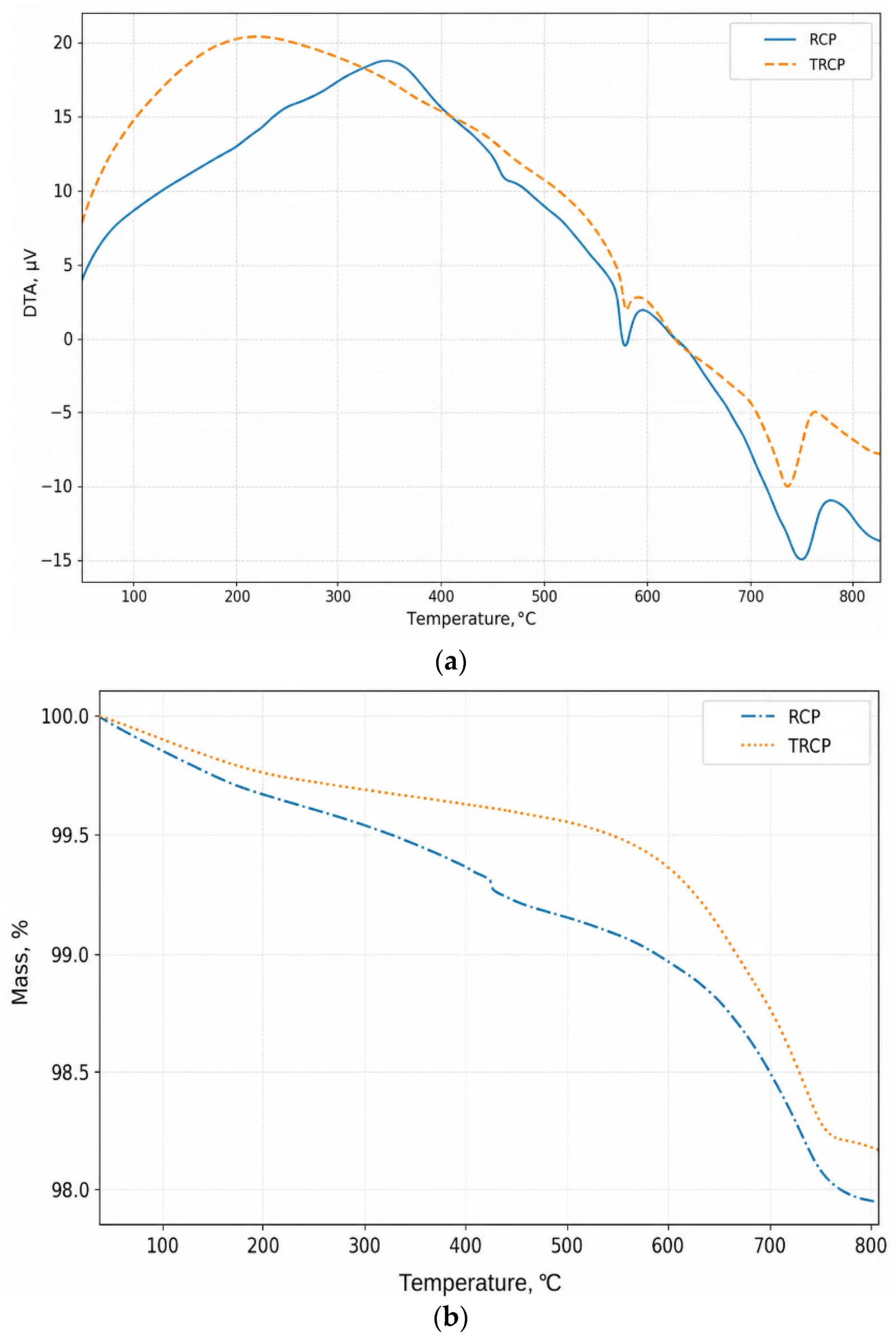
Thermoanalytical curves of untreated and thermally activated RCP: (**a**) DTA curves; (**b**) TGA curves.

For the Series II materials, DTA and TGA analyses were used to support the interpretation of phase composition and phase evolution during hardening. The DTA and TGA curves of untreated and thermally activated RCP are shown in Figure 15a,b, respectively.

The DTA curves (Figure 15a) show broad thermal effects associated with dehydration and transformation of the hydrated cement paste residues, together with pronounced high-temperature effects related mainly to carbonate decomposition. According to the TGA curves (Figure 15b), untreated RCP had a low total mass loss of about 2.04% in the range 50–800 °C, indicating the predominance of thermally stable mineral components. Nevertheless, mass losses below 200 °C, in the range 200–500 °C and at 600–800 °C show the presence of physically bound water, hydrated cement paste phases and carbonate components. After thermal activation, the total mass loss remained close (1.88%), but losses in the 105–500 °C range decreased, indicating dehydration or transformation of part of the hydrated phases during heating.

**Figure 16:**
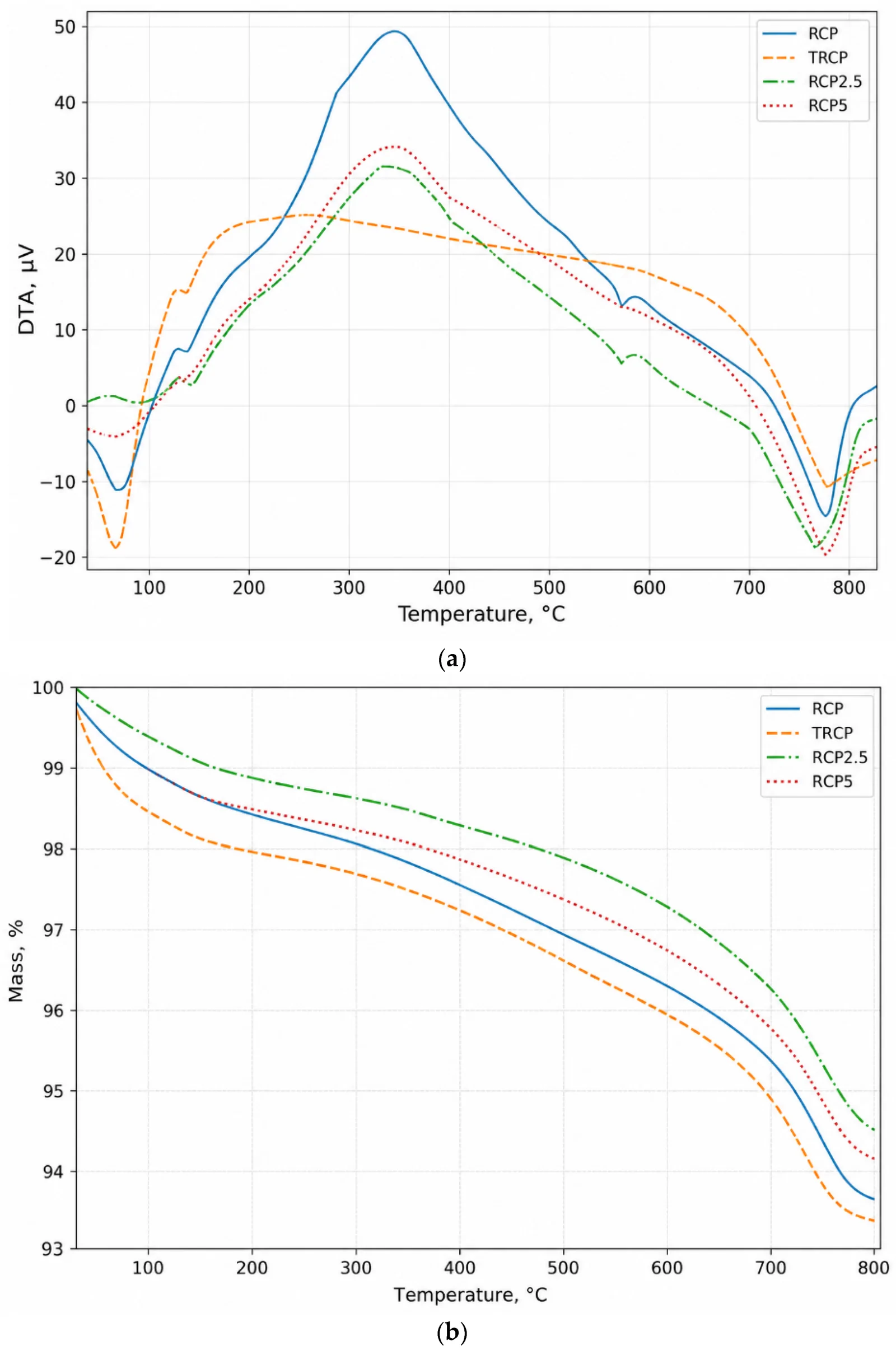
Thermoanalytical curves of hardened RCP-based pressed specimens: (**a**) DTA curves; (**b**) TGA curves.

The DTA and TGA curves of hardened pressed specimens are shown in Figure 16a,b, respectively, while the quantitative mass losses are summarized in Table 9.

As shown by the TGA curves in Figure 16b and the data in Table 9, the hardened pressed specimens had considerably higher total mass losses than the initial powders, ranging from 4.99 to 6.57%. This indicates the development of additional hydrated and carbonate-containing phases during curing. The highest total mass loss was observed for hardened RCP, while TRCP showed a lower value, consistent with the prior transformation of hydrated phases during thermal activation. For RCP2.5 and RCP5, the total mass losses of 5.88 and 6.07% reflect the formation of additional hydration products due to fresh Portland cement.

For all hardened specimens, substantial mass losses in the range 600–800 °C (2.56–3.00%) indicate a considerable content of carbonate phases. However, DTA/TGA alone cannot distinguish quantitatively between carbonates inherited from the old cement paste and those formed during curing, nor can it determine their spatial distribution within the interparticle contacts. Therefore, the thermoanalytical results are consistent with the participation of carbonate-containing phases in the hardening process but do not independently quantify the contribution of carbonation-induced bonding. Shao and Sakai [28] demonstrated such bonding under controlled carbonation of compacted RCP, while Hashmi et al. [24] reported measurable CO_2_ uptake and improved performance after accelerated carbonation of thermally activated RCP. Since accelerated carbonation was not applied in the present study, carbonate-related strengthening should be considered a plausible complementary mechanism rather than a separately verified contribution. Taken together, the DTA/TGA results and the continued strength development indicate that hardening cannot be explained by mechanical compaction alone and is consistent with residual hydration and the formation or transformation of hydrated and carbonate-containing phases.

### 3.6. Generalized Hardening Mechanism and Practical Implications

The obtained results indicate that pressed RCP-based materials form through a combined mechanism. The first stage is mechanical densification during pressing, which reduces intergranular porosity, increases contact area and provides immediate strength through friction, capillary forces and weak primary adhesion. However, the subsequent strength gain confirms that the contact zones continue to evolve during humid-air curing.

The second component is contact hardening in thin water films. Water is concentrated in narrow pores and contact zones, where it supports capillary attraction, limited dissolution, ion transport and precipitation of calcium- and silicate-containing products. The third component is residual hydration of cementitious particles preserved in RCP. The comparison of B1–B4 revealed that higher C_3_S and Ca(OH)_2_ contents and higher pH were associated with higher strength, supporting the role of residual cementitious phases.

The fourth component involves the evolution of hydrated and carbonate phases. The DTA/TGA results in Figure 15 and Figure 16 indicate that the initial powders and hardened specimens contain such phases and that curing alters their proportions. This observation is consistent with carbonation-induced interparticle bonding reported for compacted RCP [28], although that mechanism was not directly verified in the present study.

These components are supported by different levels of experimental evidence. The role of mechanical densification is directly demonstrated by the increase in density and strength with pressing pressure. The contribution of residual cementitious activity is supported by the relationship between strength development, residual C_3_S and Ca(OH)_2_ contents, pH, and curing conditions. DTA/TGA provides evidence for the presence and evolution of hydrated and carbonate-containing phases. By contrast, precipitation within individual contact zones, pore refinement, and carbonation-induced interparticle bonding were not observed directly and should be regarded as mechanistic interpretations consistent with the experimental trends and the cited literature. Further microstructural and phase-specific investigations would be required to quantify the contribution of each process.

Taken together, the Series I results demonstrate the influence of the parent concrete origin and residual mineralogical state, whereas Series II demonstrates the effects of forming conditions, thermal activation, particle-size composition, and limited Portland cement addition.

From a practical viewpoint, RCP can be considered not only as a filler or supplementary component but as the main component of pressed cement-free and low-cement mineral composites. Cement-free materials demonstrate the feasibility of this approach; thermally activated RCP provides a route to higher strength without additional clinker, and low-cement systems offer a simple way to improve strength and water resistance at very low cement contents. Further work should address product-scale forming, long-term strength, drying and shrinkage, water absorption, capillary suction, freeze–thaw resistance and durability under cyclic wetting and drying.

From an environmental and economic perspective, the three investigated routes involve different trade-offs. The use of untreated RCP avoids both thermal activation and the introduction of new Portland cement, but it provides the lowest 28-day strength and water resistance. Thermal activation increases strength without additional clinker; however, heating the powder to 600 °C and maintaining this temperature for 2 h requires thermal energy and additional processing equipment. Consequently, the absence of newly added cement does not by itself demonstrate a lower overall environmental impact or production cost. The advantages of this route will depend on furnace efficiency, production scale, transport distance, and the possibility of using waste heat or low-carbon energy. Previous studies have also shown that the performance and environmental effectiveness of thermally activated RCP depend strongly on activation temperature and its subsequent use [21,22,24].

The addition of 2.5–5 wt.% Portland cement may represent a more readily implementable alternative because it avoids furnace treatment while requiring only 25 or 50 kg of newly added Portland cement per 1000 kg of RCP. Because the cement dosage was specified relative to the RCP mass, these additions correspond to 2.44 and 4.76 wt.% of the total dry solids, respectively. These values provide a quantitative material-intensity indicator but should not be interpreted as a complete carbon-footprint or production-cost assessment. A reliable comparison with conventional cement-based products would require a functionally equivalent reference product, plant-scale electricity and fuel consumption for crushing, sieving, mixing, pressing, curing, and thermal activation, as well as transport distances, material yields, regional emission factors, and unit prices. These data were not recorded in the present laboratory study. In particular, the thermal energy required to maintain the RCP at 600 °C for 2 h may partly or completely offset the benefits associated with avoiding newly added cement. Therefore, quantitative environmental or economic superiority over conventional materials cannot yet be claimed. The compressive strengths obtained in this study, 6.9–12.8 MPa at 28 days, indicate potential for pressed products with moderate strength requirements, such as non-load-bearing masonry units or similar elements. However, direct comparison with conventional construction products remains preliminary because product-scale manufacturing, dimensional stability, long-term durability, freeze–thaw resistance, and compliance with relevant standards were not evaluated. These issues should be addressed through comparative life-cycle assessment and techno-economic analysis.

The 28-day water resistance coefficients of RCP, TRCP, RCP2.5, and RCP5 were 0.61, 0.74, 0.80, and 0.86, respectively, indicating improved compressive-strength retention after 24 h water saturation due to thermal activation and Portland cement addition. However, this coefficient is not equivalent to a water-absorption or durability test. Drying shrinkage, water absorption, and freeze–thaw resistance were not measured in the present study. Therefore, the quantitative effects of thermal activation and low-content Portland cement addition on dimensional stability and frost resistance cannot be established from the available data.

Further work should include systematic durability testing of representative RCP, TRCP, RCP2.5, and RCP5 mixtures, including drying shrinkage, water absorption, capillary suction, freeze–thaw resistance, and resistance to cyclic wetting and drying, together with product-scale forming, long-term strength evaluation, and comparative life-cycle and techno-economic assessment.

## 4. Conclusions

This study demonstrated the possibility of using recycled concrete powder as the main component for producing pressed cement-free and low-cement mineral composites. The following main conclusions can be drawn:

1. In Series I, the <0.14 mm fraction of recycled concrete powder can act as a reactive matrix component in pressed materials. Its effectiveness depends strongly on the origin of the parent concrete, particularly on residual clinker minerals, calcium hydroxide, hydrated and carbonate phases, and the alkalinity of the powder. The best RCP source in this study provided 13.4 MPa after 28 days, whereas the least active powder provided 5.6 MPa.
2. For Series II, the properties of pressed RCP-based materials are controlled by a balanced combination of forming moisture, pressure and curing conditions. For the investigated system, a forming moisture content of approximately 12–13%, a pressing pressure of 20 MPa and humid-air curing were found to be rational, providing sufficient compaction while preserving moisture required for continued hardening.
3. Within Series II, untreated RCP formed a cement-free pressed material with a 28-day compressive strength of 6.9 MPa. Thermal activation at 600 °C for 2 h increased the 28-day strength to 11.4 MPa, indicating that transformation of hydrated cement paste residues can enhance the reactivity and binding ability of old concrete powder.
4. Within Series II, small Portland cement additions improved both strength and water resistance. RCP with 2.5 wt.% Portland cement reached 10.6 MPa after 28 days, while RCP with 5 wt.% Portland cement reached 12.8 MPa and exhibited the highest water resistance coefficient of 0.86. Thus, a limited cement content can effectively stabilize the contact structure of pressed RCP-based materials.
5. For the Series II raw-material system, within the investigated factor ranges, multifraction composition modelling indicated that the properties of pressed composites depend not only on cement content but also on particle-size balance. At 2.5% cement content, the model-based rational region corresponded approximately to 36–50% of the <0.14 mm fraction, 22–35% of the 0.14–0.5 mm fraction, and 15–42% of the 0.5–1 mm fraction. This region should be regarded as an experimental–statistical estimate requiring independent validation.
6. DTA/TGA analysis indicated the presence and evolution of hydrated and carbonate-containing phases in the hardened pressed materials. Together with the mechanical, mineralogical, and pH results, these observations are consistent with a combined structure-formation mechanism involving mechanical densification, capillary and contact interactions, residual hydration, dissolution–precipitation processes, and the formation or transformation of hydrated and carbonate-containing phases. The individual contributions of these processes require further phase-specific and microstructural investigation.

## Figures and Tables

**Table 1:** Characteristics of concrete waste samples of the <0.14 mm fraction.

RCP Sample	Design Class of Parent Concrete	Approximate Age	Compressive Strength of Parent Concrete, MPa	Residue on 0.045 mm Sieve, %	Residue on 0.08 mm Sieve, %	pH
B1	C40/50	2 months	55.4	64.2	49.3	11.7
B2	C40/50	3 years	72.1	65.3	48.4	10.2
B3	C16/20	2 months	21.6	62.2	52.3	10.9
B4	C16/20	3 years	34.1	61.8	51.2	8.9

**Table 2:** X-ray diffraction data for the dispersed fraction of concrete waste samples, %.

RCP Sample	C_3_S	C_2_S	C_4_AF	Ca(OH)_2_	CaCO_3_	SiO_2_
B1	14.22	6.16	2.54	9.03	1.59	62.2
B2	7.80	1.79	2.83	7.32	6.63	68.7
B3	7.25	1.42	3.69	8.76	2.39	72.3
B4	0.22	3.51	0.27	2.41	4.89	84.4

**Table 3:** Chemical and mineralogical composition of the initial recycled concrete powder.

Chemical Composition, wt.%	Value	Mineralogical Composition, %	Value
CaO	13.2	Quartz	50.5
SiO_2_	75.5	Feldspars	22.7
Al_2_O_3_	6.28	Calcite	10.9
Fe_2_O_3_	3.56	C_3_S	0.86
MgO	0.61	C_2_S	2.09
—		Hydrated cement products	14.1

The dash has been retained to indicate that there is no corresponding additional component in the chemical composition column, because the chemical and mineralogical compositions contain different numbers of reported components.

**Table 4:** Properties of pressed composites prepared from different recycled concrete powder samples. Values are presented as mean ± standard deviation (*n* = 3).

Concrete Sample	Compressive Strength, MPa, at 1 h	Compressive Strength, MPa, at 1 Day	Compressive Strength, MPa, at 7 Days *	Compressive Strength, MPa, at 28 Days *	Dry Bulk Density, g/cm^3^	Water Resistance Coefficient, 28 Days
B1	3.1 ± 0.17	4.6 ± 0.22	11.5 ± 0.78	13.4 ± 0.64	1.62 ± 0.03	0.92 ± 0.03
B2	2.9 ± 0.19	3.8 ± 0.17	6.7 ± 0.38	8.7 ± 0.60	1.59 ± 0.03	0.87 ± 0.03
B3	3.2 ± 0.16	4.1 ± 0.15	8.6 ± 0.54	10.2 ± 0.58	1.61 ± 0.03	0.91 ± 0.04
B4	3.0 ± 0.16	3.5 ± 0.22	4.2 ± 0.28	5.6 ± 0.32	1.56 ± 0.02	0.88 ± 0.03

* Specimens were stored above water.

**Table 5:** Physical and mechanical properties of RCP-based pressed materials at different curing ages. Values are presented as mean ± standard deviation (*n* = 3).

Material	Compressive Strength, MPa			Bulk Density, g/cm^3^			Water Resistance Coefficient		
	3 Days	7 Days	28 Days	3 Days	7 Days	28 Days	3 Days	7 Days	28 Days
RCP	4.3 ± 0.24	5.1 ± 0.20	6.9 ± 0.43	1.64 ± 0.03	1.66 ± 0.02	1.69 ± 0.03	0.50 ± 0.02	0.54 ± 0.02	0.61 ± 0.02
TRCP	4.6 ± 0.19	6.8 ± 0.26	11.4 ± 0.58	1.63 ± 0.03	1.67 ± 0.02	1.71 ± 0.02	0.52 ± 0.02	0.60 ± 0.02	0.74 ± 0.02
RCP2.5	5.4 ± 0.34	7.8 ± 0.48	10.6 ± 0.53	1.66 ± 0.02	1.70 ± 0.03	1.74 ± 0.02	0.61 ± 0.02	0.72 ± 0.02	0.80 ± 0.02
RCP5	6.5 ± 0.40	9.4 ± 0.53	12.8 ± 0.79	1.68 ± 0.03	1.72 ± 0.02	1.76 ± 0.03	0.69 ± 0.02	0.78 ± 0.02	0.86 ± 0.03

**Table 6:** Experimental design conditions.

No.	Factor	Code	Natural	−1	0	+1	Variation Interval
1	Fine fraction content	X_1_	Fraction < 0.14 mm, %	20	35	50	15
2	Medium fraction content	X_2_	Fraction 0.14–0.5 mm, %	15	25	35	10
3	Portland cement content	X_3_	Portland cement, %	0	2.5	5.0	2.5

**Table 7:** Experimental results for the properties of pressed composites. Values are presented as mean ± standard deviation (*n* = 3).

No.	x_1_	x_2_	x_3_	<0.14 mm, %	0.14–0.5 mm, %	0.5–1 mm, %	PC, %	f_cm_, MPa	WRC
1	+1	+1	+1	50	35	15	5.0	11.5 ± 0.71	0.84 ± 0.03
2	+1	+1	−1	50	35	15	0	7.7 ± 0.30	0.67 ± 0.02
3	+1	−1	+1	50	15	35	5.0	10.9 ± 0.61	0.81 ± 0.03
4	+1	−1	−1	50	15	35	0	5.4 ± 0.30	0.57 ± 0.01
5	−1	+1	+1	20	35	45	5.0	8.8 ± 0.54	0.79 ± 0.02
6	−1	+1	−1	20	35	45	0	5.9 ± 0.25	0.56 ± 0.02
7	−1	−1	+1	20	15	65	5.0	8.1 ± 0.49	0.68 ± 0.02
8	−1	−1	−1	20	15	65	0	5.6 ± 0.30	0.57 ± 0.02
9	+1	0	0	50	25	25	2.5	9.9 ± 0.48	0.75 ± 0.02
10	−1	0	0	20	25	55	2.5	7.1 ± 0.38	0.66 ± 0.02
11	0	+1	0	35	35	30	2.5	8.5 ± 0.54	0.74 ± 0.03
12	0	−1	0	35	15	50	2.5	8.9 ± 0.54	0.66 ± 0.02
13	0	0	+1	35	25	40	5.0	10.4 ± 0.48	0.79 ± 0.03
14	0	0	−1	35	25	40	0	7.1 ± 0.40	0.64 ± 0.02
15	0	0	0	35	25	40	2.5	9.3 ± 0.58	0.72 ± 0.02
16	0	0	0	35	25	40	2.5	8.6 ± 0.52	0.73 ± 0.03
17	0	0	0	35	25	40	2.5	9.1 ± 0.46	0.73 ± 0.02

**Table 8:** Regression models and coefficients of determination for the properties of pressed composites.

Response Parameter	Mathematical Model	R^2^	Adjusted R^2^
Compressive strength, MPa	f_cm_ = 8.99 + 0.99x_1_ + 0.35x_2_ + 1.80x_3_ + 0.24x_1_x_2_ + 0.49x_1_x_3_ − 0.16x_2_x_3_ − 0.49x_1_^2^ − 0.29x_2_^2^ − 0.24x_3_^2^	0.960	0.908
Water resistance coefficient, WRC	WRC = 0.725 + 0.038x_1_ + 0.031x_2_ + 0.089x_3_ + 0.004x_1_x_2_ + 0.008x_1_x_3_ + 0.006x_2_x_3_ − 0.016x_1_^2^ − 0.018x_2_^2^ − 0.005x_3_^2^	0.951	0.888

**Table 9:** Mass losses in characteristic temperature intervals according to TGA data, %.

Material	Sample Age/State	50–800 °C	50–105 °C	105–200 °C	200–350 °C	350–500 °C	500–600 °C	600–800 °C
RCP	Initial powder	2.04	0.15	0.21	0.23	0.32	0.18	0.97
TRCP	Initial powder after thermal activation	1.88	0.14	0.16	0.10	0.10	0.22	1.17
RCP	Hardened specimen, 28 days	6.57	1.07	0.63	0.45	0.73	0.68	3.00
TRCP	Hardened specimen, 28 days	4.99	1.46	0.44	0.18	0.16	0.20	2.56
RCP2.5	Hardened specimen, 28 days	5.88	0.52	0.63	0.44	0.64	0.69	2.97
RCP5	Hardened specimen, 28 days	6.07	0.70	0.64	0.43	0.65	0.69	2.96

## Data Availability

The original contributions presented in this study are included in the article. Further inquiries can be directed to the corresponding author.
